# DARPP-32 and t-DARPP promote non-small cell lung cancer growth through regulation of IKKα-dependent cell migration

**DOI:** 10.1038/s42003-018-0050-6

**Published:** 2018-05-03

**Authors:** Sk. Kayum Alam, Matteo Astone, Ping Liu, Stephanie R. Hall, Abbygail M. Coyle, Erin N. Dankert, Dane K. Hoffman, Wei Zhang, Rui Kuang, Anja C. Roden, Aaron S. Mansfield, Luke H. Hoeppner

**Affiliations:** 1 The Hormel Institute, University of Minnesota, Austin, MN 55912 USA; 2 Department of Biochemistry and Molecular Biology, Mayo Clinic, Rochester, MN 55905 USA; 3 Department of Oncology, The Second Xiangya Hospital of Central South University, Changsha, Hunan China; 4 Department of Computer Science and Engineering, University of Minnesota, Minneapolis, MN 55455 USA; 5 Department of Laboratory Medicine and Pathology, Mayo Clinic, Rochester, MN 55905 USA; 6 Department of Oncology, Division of Medical Oncology, Mayo Clinic, Rochester, MN 55905 USA

## Abstract

Lung cancer is the leading cause of cancer-related death worldwide. Here we demonstrate that elevated expression of dopamine and cyclic adenosine monophosphate-regulated phosphoprotein, Mr 32000 (DARPP-32), and its truncated splice variant t-DARPP promote lung tumor growth, while abrogation of DARPP-32 expression in human non-small-cell lung cancer (NSCLC) cells reduces tumor growth in orthotopic mouse models. We observe a physical interaction between DARPP-32 and inhibitory kappa B kinase-α (IKKα) that promotes NSCLC cell migration through non-canonical nuclear factor kappa-light-chain-enhancer of activated B cells 2 (NF-κB2) signaling. Bioinformatics analysis of 513 lung adenocarcinoma patients reveals that elevated t-DARPP isoform expression is associated with poor overall survival. Histopathological investigation of 62 human lung adenocarcinoma tissues also shows that t-DARPP expression is elevated with increasing tumor (T) stage. Our data suggest that DARPP-32 isoforms serve as a negative prognostic marker associated with increasing stages of NSCLC and may represent a novel therapeutic target.

## Abstract (web summary)

Sk. Kayum Alam et al. show that DARPP-32 isoforms, mediators of dopamine signaling, promote lung tumor growth through non-canonical NF-κB signaling. This study suggests a possibility of using DARPP-32 isoforms as a prognostic marker for lung cancer.

## Introduction

Lung cancer is the leading cause of cancer deaths among both men and women^1^. In 2017, an estimated 160,420 lung cancer deaths will occur in the United States^2^. Non-small-cell lung cancer (NSCLC) represents 85–90% of all cases of lung cancer and carries a very poor survival rate with less than 15% of patients surviving more than 5 years^3,4^. Despite administration of standard chemotherapeutic agents with evolving systemic cancer therapies directed at driver mutations (epidermal growth factor receptor (EGFR), BRAF and ALK), inhibiting angiogenesis (anti-vascular endothelial growth factor therapy) and immune-checkpoint blockade (anti-programmed death-1 antibody), these statistics remain dismal due to the large number of patients diagnosed with advanced-stage disease and the primary and secondary resistance to current therapies. A better understanding of the mechanisms that regulate lung tumor growth, metastasis and drug resistance will result in new diagnostic tools and therapeutic strategies to improve the clinical outlook and quality of life of patients afflicted with this deadly disease.

Dopamine and cyclic adenosine monophosphate-regulated phosphoprotein, Mr 32000 (DARPP-32), is an effector molecule that plays an important role in dopaminergic neurotransmission. This 32 kDa protein was initially discovered in the neostriatum in the brain as substrate of dopamine-activated protein kinase A (PKA)^5^. Phosphorylation at threonine-34 (T34) by PKA causes DARPP-32-mediated inhibition of protein phosphatase-1 (PP-1)^6^, hence DARPP-32 is also called *phosphoprotein phosphatase-1 regulatory subunit 1B* (*PPP1R1B*). DARPP-32 is converted to an inhibitor of PKA upon phosphorylation of its T75 residue by cyclin-dependent kinase 5 (Cdk5)^7^. The ability of DARPP-32 to function as either a kinase or a phosphatase inhibitor enables it to precisely modulate dopaminergic neurotransmission^7,8^.

In the early 2000s, El-Rifai and colleagues^9,10^ discovered DARPP-32 is frequently amplified and upregulated in gastric cancer. Cloning and sequence assembly analysis revealed a transcriptional splice variant of DARPP-32 is also overexpressed in gastric cancer. The N-terminally truncated isoform of DARPP-32, termed t-DARPP, was found to utilize a unique alternative first exon located within intron 1 of DARPP-32 and to lack the first 36 amino acids of DARPP-32, including the T34 phosphorylation residue required for DARPP-32-mediated PP-1 inhibition^9^. Overexpression of both DARPP-32 and t-DARPP has been observed in 68% of gastric cancers^9,10^. Elevated expression levels of DARPP-32 and t-DARPP have also been associated with many adenocarcinomas, including stomach, colon, prostate and breast cancers^11–16^. Reports have implicated DARPP-32 and t-DARPP in cancer cell proliferation, survival, invasion and angiogenesis^17^. Several studies have demonstrated that DARPP-32 and t-DARPP protect cancer cells from drug-induced apoptosis, which is dependent upon their T75 phosphorylation residue^9,10^ and involves upregulation of Akt and Bcl2 proteins^16,18,19^. Most recently, t-DARPP overexpression was shown to promote activation of insulin-like growth factor-1 receptor signaling to regulate glucose metabolism as a mechanism of trastuzumab resistance in breast cancer cells^20^. To date, the role of DARPP-32 isoforms in lung cancer remains unexplored. However, we recently described the role of dopamine signaling in NSCLC by demonstrating that dopamine D2 receptor agonists inhibit lung cancer growth by reducing angiogenesis and tumor-infiltrating myeloid-derived suppressor cells in preclinical orthotopic murine models^21^. Given the role of dopamine signaling in lung cancer and the oncogenic nature of DARPP-32 isoforms in a variety of tumor types, we sought to determine whether DARPP-32 and t-DARPP contribute to lung cancer growth, progression and drug resistance.

Nuclear factor kappa-light-chain-enhancer of activated B cells (NF-κB) is a transcription factor that regulates numerous biological processes, such as immunity, inflammation, cell growth, differentiation, migration, tumorigenesis and apoptosis^22^. The family of NF-κB proteins is comprised of structurally homologous transcription factors, including NF-κB1 (p105/50), NF-κB2 (p100/52), RelA (p65), RelB and c-Rel^23^. In the absence of external stimuli, NF-κB proteins are sequestered in the cytoplasm by specific inhibitory proteins, inhibitors of NF-κB (IκBs)^22^. When a cell receives appropriate stimuli, IκB kinase (IKK) phosphorylation is initiated, leading to proteasome-mediated processing of p105 and p100. This cleavage event generates their respective mature proteins, p50 and p52, resulting in the nuclear translocation of previously sequestered NF-κB members^24^. NF-κB signaling has been categorized into canonical and non-canonical (i.e., alternative) pathways. Recent studies have shown that both canonical and non-canonical NF-κB pathways are capable of promoting oncogenesis by interacting with other cellular pathways in breast cancer, pancreatic ductal adenocarcinoma (PDAC) and glioblastomas^25–27^. NF-κB1 pathway activation causes induction of the IKK complex that contains two catalytic subunits, IKKα and IKKβ, and one scaffold subunit called nuclear factor κB essential modulator (NEMO) or IKKγ^28,29^. Dysregulation of the IKK complex can initiate constitutive activation of the NF-κB1 pathway in cancer cells^30^. Non-canonical NF-κB2 signaling requires IKKα to mediate p100 cleavage into p52, but does not depend upon IKKβ and NF-κB essential modulator (NEMO), which are essential for canonical NF-κB1 signal transduction^31,32^. A recent finding has suggested that constitutive activation of KRAS and IKK/NF-κB1 pathways expedites tumorigenesis and worsens survival in PDAC patients^33^. Ablation of constitutive IKK activity by small molecule inhibitor reduces cellular NF-κB1 activity and melanoma cell survival in vitro and in vivo^34^. A recent report has suggested that proinflammatory *Helicobacter pylori* infection and canonical NF-κB1 activation play an important role in the regulation of DARPP-32 expression, which has been shown to counteract infection-induced cell death and promote cell survival in gastric carcinogenesis^35^.

We aimed to investigate the role of DARPP-32 isoforms in NSCLC. Here we demonstrate that DARPP-32 and t-DARPP promote cell survival and non-canonical NF-κB2 p52-mediated cell migration in lung cancer. In NSCLC patients, elevated expression of t-DARPP was found to be associated with tumor stage and worsened patient survival.

## Results

### DARPP-32 and t-DARPP promote NSCLC cell survival via Akt/Erk signaling

**Fig. 1:**
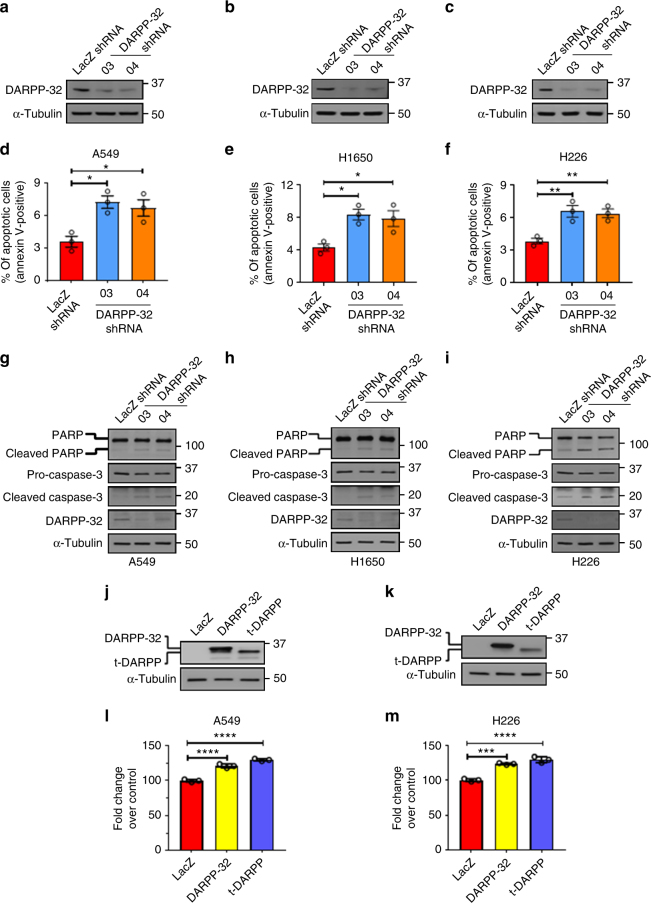
DARPP-32 promotes cell survival and negatively regulates apoptosis. **a** NSCLC A549, **b** H1650 and **c** H226 cell lines were transduced with lentivirus encoding LacZ control shRNA or DARPP-32 shRNAs (clone numbers 03 and 04). DARPP-32 and α-tubulin (loading control) proteins were detected by immunoblotting of cell lysates. Immunoblots are representative of three independent experiments. **d** A549, **e** H1650 and **f** H226 cells transduced with control or DARPP-32 shRNAs were seeded into 60 mm culture dishes for 16 h. Flow cytometry-based apoptosis assays were performed following incubation with anti-annexin V antibodies conjugated with APC. **g** A549, **h** H1650 and **i** H226 cells were transduced with control or DARPP-32 shRNAs and immunoblotted with antibodies to detect cleaved and uncleaved PARP, cleaved and uncleaved (i.e., pro-) caspase-3, DARPP-32 and α-tubulin (loading control). **j** A549 and **k** H226 cells were transduced with retrovirus containing control (LacZ), DARPP-32- or t-DARPP-overexpressing clones. DARPP-32, t-DARPP and α-tubulin (loading control) proteins were detected by immunoblotting of cell lysates. Immunoblots are representative of three independent experiments. **l** A549 and **m** H226 cells transduced with control, DARPP-32- or t-DARPP-overexpressing clones were seeded into 96-well cell culture plates for 72 h. Colorimeter-based cell survival assay was conducted using MTS reagents. Each open circle on a graph represents an independent experiment. Uncropped images of depicted immunoblots are shown in Supplementary Figs. 13-15. Error bars indicate SEM (*n* = 3). *P < 0.05, **P < 0.01, ***P < 0.001 and ****P < 0.0001, one-way ANOVA followed by Dunnett’s test for multiple comparison

Given the oncogenic role of DARPP-32 in gastric and breast cancer progression^10,12,36^, we sought to determine whether DARPP-32 proteins regulate cell survival in NSCLC. First, we stably silenced endogenous DARPP-32 protein expression through lentiviral short hairpin RNA (shRNA)-mediated knockdown in A549 and H1650 human lung adenocarcinoma cells as well as H226 human lung squamous cell carcinoma cells (Fig. 1a–c). Two shRNAs targeting distinct regions of DARPP-32 were utilized to decrease the likelihood of potentially confounding off-target effects (Fig. 1a–c). To determine the role of DARPP-32 in regulation of cell survival, we first assessed apoptosis upon DARPP-32 knockdown using flow cytometry-based annexin V assays and detection of apoptosis-associated proteins by immunoblotting. We observed increased annexin V-positive cells, along with elevated expression of cleaved poly(ADP-ribose) polymerase (PARP) and caspase-3 proteins, in DARPP-32 knockdown cell lines compared to controls (Fig. 1d–i), suggesting that DARPP-32 inhibits apoptosis in lung cancer cells. We also performed annexin V assays and immunoblotting in A549, H1650 and H226 cell lines overexpressing DARPP-32 isoforms. An N-terminally truncated isoform and transcriptional variant of DARPP-32, called t-DARPP, lacks the protein phosphate inhibitory (PP-1) domain, which is phosphorylated at T34 and important for dopamine signaling function^9^. Apoptosis was decreased in DARPP-32- and t-DARPP-overexpressing cells compared to corresponding LacZ-transduced controls based on decreased annexin V, cleaved PARP and caspase-3 proteins (Supplementary Fig. 1a, b, c, d). Based on this finding, we next performed a colorimetric cell viability assay in A549 and H226 cells stably transduced with retrovirus to overexpress exogenous DARPP-32 and t-DARPP proteins (Fig. 1j, k). Cell viability was increased in DARPP-32-overexpressing cells compared to corresponding LacZ-transduced controls (Fig. 1l, m). Overexpression of t-DARPP in A549 and H226 lung cancer cells increased viability (Fig. 1l, m), suggesting that the N-terminal T34-dependent PP-1 regulatory function of DARPP-32^37^ does not contribute to regulation of cell viability. Given the role of t-DARPP in promoting cellular proliferation in gastrointestinal cancer^38^, we sought to determine whether DARPP-32 and t-DARPP proteins regulate proliferation of NSCLC cells. We found modulation of DARPP-32 isoforms does not alter proliferation of lung cancer cells using flow cytometry-based bromodeoxyuridine (BrdU) cell proliferation assays upon silencing endogenous DARPP-32 and overexpression of DARPP-32 and t-DARPP (Supplementary Fig. 2). Taken together, our findings suggest that DARPP-32 and t-DARPP promote lung tumor cell survival by regulating apoptosis but do not control cellular proliferation.

**Fig. 2:**
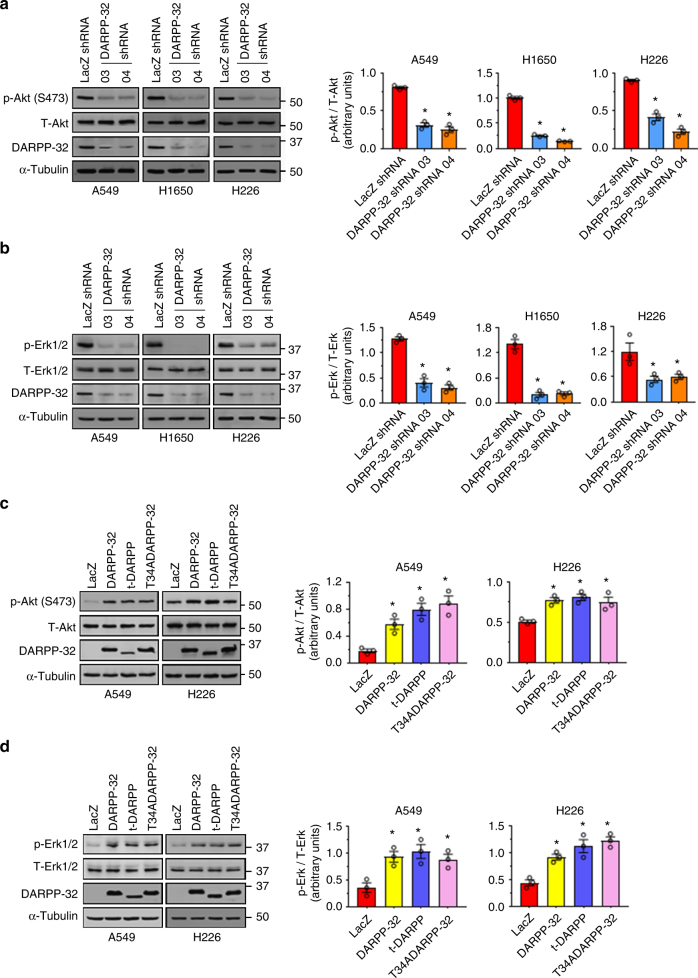
DARPP-32 regulates cell survival through Akt and Erk1/2. **a** A549, H1650 and H226 cells were transduced with control or DARPP-32 shRNAs. Cell lysates were collected and immunoblotted with antibodies against phosphorylated Akt (p-Akt; S473), total Akt (T-Akt), **b** phosphorylated Erk1/2 (p-Erk1/2), total Erk1/2 (T-Erk1/2), DARPP-32 and α-tubulin (loading control). **c** A549 and H226 cells were transduced with control, DARPP-32-, t-DARPP- or T34A DARPP-32-overexpressing clones. Phosphorylated Akt (p-Akt; S473), total Akt (T-Akt), **d** phosphorylated Erk1/2 (p-Erk1/2), total Erk1/2 (T-Erk1/2), DARPP-32 and α-tubulin (loading control) proteins were detected by immunoblotting of cell lysates. Densitometry of the indicated immunoblots was performed using ImageJ software. Each open circle on a graph represents an independent experiment. All immunoblots are representative of three independent experiments. Uncropped images of depicted immunoblots are shown in Supplementary Figs. 16, 17. All bar graphs represent mean ± SEM (*n* = 3). *P < 0.05, one-way ANOVA followed by Dunnett’s test for multiple comparison

To elucidate the molecular mechanism through which DARPP-32 proteins control NSCLC cell survival, we investigated Akt and extracellular signal–regulated kinase-1/2 (Erk1/2) signaling as both pathways have been previously implicated in DARPP-32-mediated regulation of cell survival^12,18,21^. Ablation of DARPP-32 decreased phosphorylated Akt (p-Akt) and phosphorylated Erk (p-Erk) levels substantially, while corresponding total Akt and Erk1/2 protein expression remained unchanged by immunoblotting (Fig. 2a, b). Correspondingly, DARPP-32 overexpression resulted in increased phosphorylation of Akt and Erk1/2 (Fig. 2c, d). We found exogenous overexpression of t-DARPP and mutant DARPP-32 (T34A) proteins also elevates p-Akt and p-Erk1/2 levels, suggesting PP-1 activation by DARPP-32 T34 phosphorylation is not directly involved in stimulation of Akt and Erk signaling (Fig. 2c, d). Collectively, our results suggest that DARPP-32 promotes cell survival in a PP-1-independent manner through Akt and Erk1/2 signaling in NSCLC cells.

### DARPP-32 promotes lung cancer cell migration

**Fig. 3:**
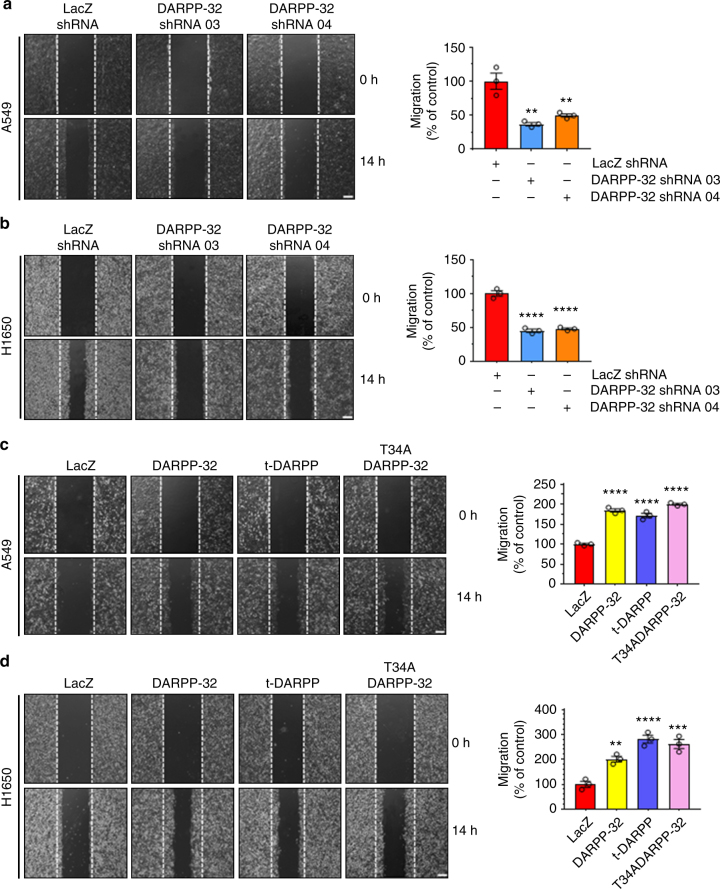
DARPP-32 positively regulates cell migration. **a** A549 and **b** H1650 cells transduced with lentivirus encoding control or DARPP-32 shRNAs were plated into 60 mm cell culture dishes, scratched and imaged at 0 and 14 h. **c** A549 and **d** H1650 cells infected with retrovirus encoding control, DARPP-32, t-DARPP or T34A DARPP-32 clones were scratched and imaged at 0 and 14 h. Dashed lines indicate the boundary of the edges of the wound at 0 h. Experiments were repeated at least three times in triplicate. Scale bar, 200 µm. Distance traveled by the migratory cells were calculated using ImageJ software. Each open circle on a graph represents an independent experiment. Results represent mean ± SEM (*n* = 3). **P < 0.01, ***P < 0.001 and ****P < 0.0001, one-way ANOVA followed by Dunnett’s test for multiple comparison

DARPP-32 is upregulated in various cancers including breast and gastric cancer, in which expression of DARPP-32 is associated with increased migration and invasion^39,40^. To determine the role of DARPP-32 in NSCLC motility, we performed scratch wound healing assays using A549 and H1650 lung adenocarcinoma cells. We observed a substantial decrease in cellular migration of DARPP-32 shRNA-silenced A549 and H1650 cells compared to controls (Fig. 3a, b). We next investigated whether overexpression of DARPP-32 enhances cell motility in lung cancer cells. DARPP-32, as well as t-DARPP and the T34A DARPP-32 mutant, promoted increased migration in A549 and H1650 cells (Fig. 3c, d). To validate this result using a more physiologically relevant three-dimensional culture system, we performed Matrigel spot assays to assess lung tumor cell migration. A549 and H1650 lung adenocarcinoma cells stably transduced with lentivirus encoding control or DARPP-32 shRNAs were mixed with Matrigel and spotted on a cell culture plate followed by addition of medium. Similar to our previous findings, DARPP-32 knockdown substantially decreased tumor cell migration in A549 and H1650 cells (Supplementary Fig. 3a, b). Moreover, A549 and H1650 cells stably overexpressing DARPP-32, t-DARPP or mutant DARPP-32 (T34A) increased cell migration compared to control (Supplementary Fig. 4a, b). Taken together, our results suggest DARPP-32 promotes lung tumor cell migration.

### DARPP-32 interacts with IKKα to activate non-canonical NF-ĸB2 signaling

**Fig. 4:**
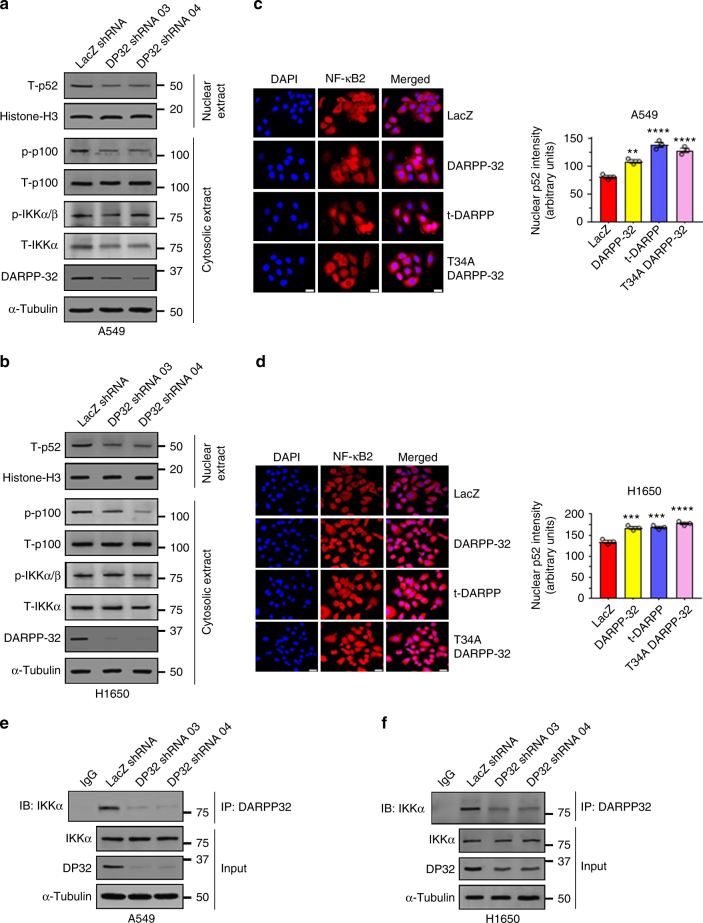
DARPP-32 knockdown inhibits non-canonical NF-ĸB2 signaling. **a** Nuclear fractions of A549 and **b** H1650 cells expressing control or DARPP-32 (DP32) shRNAs were immunoblotted with antibodies against total p52 (T-p52) and histone H3 (loading control). Cytosolic fractions were also collected and subjected to western blotting using antibodies against phosphorylated p100 (p-p100), total p100 (T-p100), phosphorylated IKKα/β (p-IKKα/β), total IKKα (T-IKKα), DARPP-32 and α-tubulin (loading control). **c** Immunofluorescence studies were performed using a monoclonal NF-ĸB2 antibody (that detects both p100 and p52 proteins) on A549 and **d** H1650 cell lysates expressing control or DARPP-32 shRNAs. Nuclei were labeled with DAPI. Average red fluorescence intensity of all nuclei was calculated using ImageJ software. Experiments were repeated at least three times. Each open circle on a graph represents an independent experiment. Scale bar, 20 µm. Error bars indicate SEM (*n* = 3). **P < 0.01, ***P < 0.001 and ****P < 0.0001, one-way ANOVA followed by Dunnett’s test for multiple comparison. **e** A549 and **f** H1650 cells transduced with lentivirus encoding control or DARPP-32 shRNAs (DP32 shRNAs) were immunoprecipitated (IP) with anti-DARPP-32 antibody and immunoblotted (IB) with antibody against IKKα. Total cell lysates (Input) were subjected to western blotting using antibodies against IKKα, DARPP-32 (DP32) and α-tubulin (loading control). All immunoblots are representative of three independent experiments. Uncropped images of depicted immunoblots are shown in Supplementary Figs. 18, 19

We sought to determine the molecular signaling through which DARPP-32 promotes migration. Lung tumor cell migration has been previously shown to be regulated by non-canonical NF-ĸB2 signaling^41^. Thus, we hypothesized that DARPP-32 stimulates cell migration through modulation of non-canonical NF-ĸB2 signaling. In stimulated cells, NF-ĸB-inducing kinase (NIK) activates inhibitory kappa B kinase-α (IKKα) which, in turn, phosphorylates cytosolic NF-ĸB2 p100 causing its cleavage to NF-ĸB2 p52, which translocates to the nucleus to transcriptionally regulate gene expression^42^. By immunoblotting, we found DARPP-32 ablation in A549 and H1650 human lung adenocarcinoma cells reduced the NF-ĸB2 p52 to p100 ratio, suggesting DARPP-32 positively regulates non-canonical NF-ĸB2 signaling (Supplementary Fig. 5a, b). Correspondingly, we found DARPP-32 knockdown decreased cytosolic phosphorylated NF-ĸB2 p100 and nuclear NF-ĸB2 p52 protein expression in A549 and H1650 cells (Fig. 4a, b). Given our immunoblotting result suggesting DARPP-32 promotes nuclear p52 expression, we sought to determine whether elevated DARPP-32 increases nuclear NF-ĸB2 p52 localization using immunofluorescence. Indeed, we observed greater nuclear localization of p52 in A549 and H1650 lung cancer cells overexpressing DARPP-32, t-DARPP or DARPP-32 T34A relative to controls (Fig. 4c, d). In accordance with these results, western blot data confirmed that overexpression of DARPP-32 activates NF-κB2 signaling by increasing the expression of cytosolic phosphorylated p100 and nuclear p52 protein (Supplementary Fig. 6). Interestingly, knockdown (Fig. 4a, b) or overexpression (Supplementary Fig. 6b, d) of DARPP-32 had no effect on phosphorylation of cytosolic IKKα, suggesting activation of NF-κB2 signaling is regulated by DARPP-32 in an NIK-independent manner. Thus, we sought to determine whether DARPP-32 is capable of activating NF-κB2 signaling in an NIK-independent manner through a direct interaction with IKKα. We demonstrate a physical association between DARPP-32 and IKKα through co-immunoprecipitation studies in A549 and H1650 human lung adenocarcinoma cells (Fig. 4e, f). The interaction of DARPP-32 and IKKα was substantially decreased upon DARPP-32 ablation (Fig. 4e, f). To determine whether DARPP-32 isoforms regulate transcriptional effectors of non-canonical NF-ĸB2 signaling, we assessed the messenger RNA (mRNA) expression of *EZH2*^43^ and *BIRC3*^44^ transcriptional targets upon DARPP-32 isoform modulation in A549 and H1650 human NSCLC cells. We observed decreased expression of *EZH2* and *BIRC3* transcripts upon knockdown of DARPP-32 (Supplementary Fig. 7a, b, c, d), whereas these NF-ĸB2 signaling targets increased upon overexpression of DARPP-32 or t-DARPP in NSCLC cells relative to controls (Supplementary Fig. 7e, f, g, h). Taken together, our findings suggest that DARPP-32 activates non-canonical NF-κB2 signaling by interacting with IKKα.

### DARPP-32 stimulates cell migration via nuclear translocation of NF-κB2 p52

**Fig. 5:**
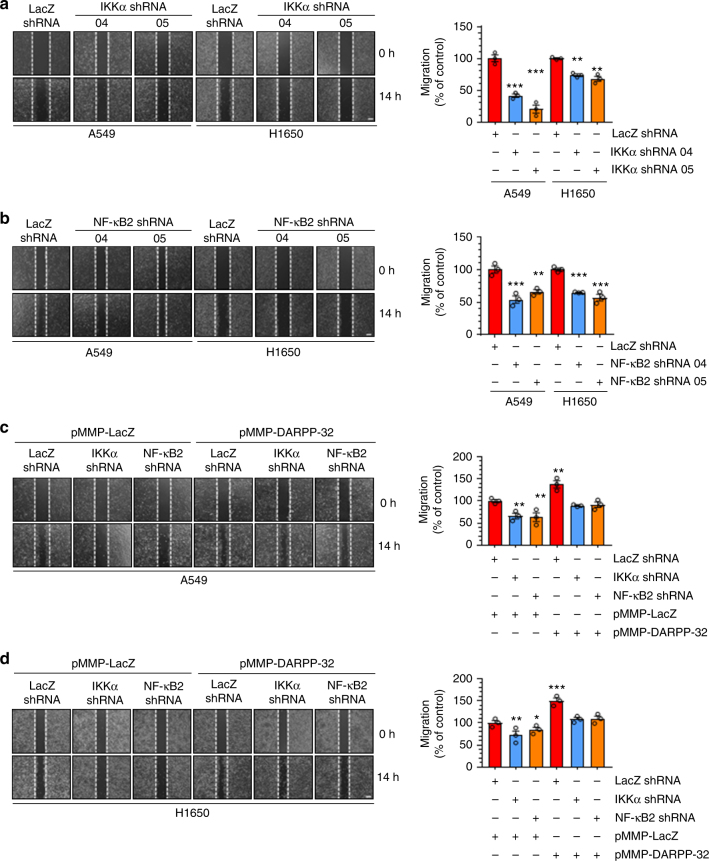
Knockdown of IKKα and NF-ĸB2 reduces lung cancer cell migration. **a** A549 and H1650 cells transduced with lentivirus encoding control or IKKα shRNAs (clone numbers 04 and 05) were plated into 60 mm cell culture dishes, scratched and imaged at 0 and 14 h. **b** A549 and H1650 cells transduced with lentivirus encoding control or NF-ĸB2 shRNAs (clone numbers 04 and 05) were scratched and imaged at 0 and 14 h. **c** A549 and **d** H1650 cells expressing control, IKKα or NF-κB2 shRNAs were transfected with control (pMMP-LacZ) or DARPP-32 (pMMP-DARPP-32) overexpressing plasmids using Polyfect reagent. The cells were then scratched and imaged at 0 and 14 h. Dashed lines indicate the boundary of the edges of the wound at 0 h. Experiments were repeated at least three times in triplicate. Each open circle on a graph represents an independent experiment. Scale bar, 200 µm. Distance traveled by the migratory cells were calculated using ImageJ software. Results represent mean ± SEM (*n* = 3). *P < 0.05, **P < 0.01 and ***P < 0.001, one-way ANOVA followed by Dunnett’s test for multiple comparison

We next aimed to further investigate how DARPP-32 and IKKα regulate cell migration. The IKKα-dependent non-canonical NF-ĸB2 pathway has a well-documented role in cell motility^45^. To confirm the role of IKKα in the non-canonical NF-ĸB2-mediated regulation of tumor cell migration, we performed the scratch wound healing assay with control or IKKα-depleted NSCLC cells (Fig. 5a and Supplementary Fig. 8a, b). We observed a substantial decrease in migration of IKKα shRNA-transduced cells compared to those expressing control shRNA (Fig. 5a). Next, we examined migration in A549 and H1650 cells upon shRNA-mediated NF-ĸB2 knockdown (Fig. 5b and Supplementary Fig. 8c, d). NF-κB2 depletion decreased tumor cell migration in the scratch wound healing assay (Fig. 5b), suggesting both IKKα and NF-κB2 proteins are potent activators of lung tumor cell migration. Based on our cumulative findings that DARPP-32 regulates migration (Fig. 3) and activates non-canonical NF-κB2 signaling (Fig. 4), we hypothesized that DARPP-32 stimulates cell migration through IKKα and NF-ĸB2 signaling. To test whether DARPP-32 requires downstream IKKα and NF-κB2 signaling to promote migration, we overexpressed DARPP-32 upon shRNA-mediated knockdown of IKKα or NF-ĸB2 in human NSCLC cells (Supplementary Fig. 9a, b). Migration was not altered in IKKα- or NF-ĸB2-depleted NSCLC cells upon overexpression of DARPP-32, but migration was substantially increased when DARPP-32 was overexpressed in the absence of IKKα or NF-ĸB2 knockdown (Fig. 5c, d). Thus, our findings suggest that DARPP-32 acts specifically through IKKα and NF-ĸB2 signaling to induce lung tumor migration (Supplementary Fig. 10).

### DARPP-32 promotes lung tumor growth in orthotopic murine models

**Fig. 6:**
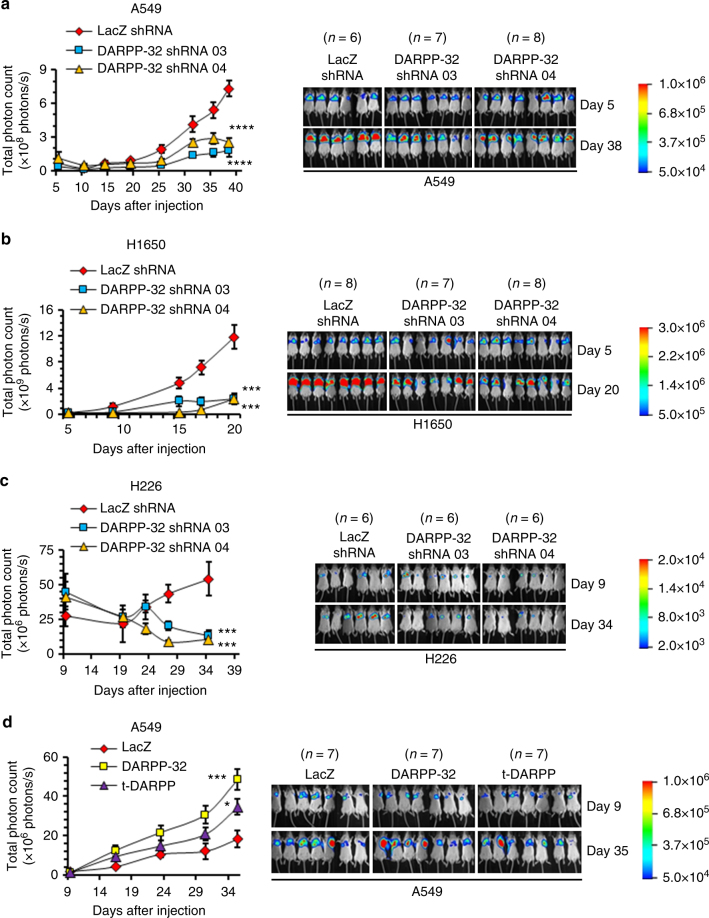
DARPP-32 knockdown decreases tumor progression in a human lung tumor xenograft model. **a** Luciferase-labeled human A549, **b** H1650 and **c** H226 cells transduced with lentivirus encoding control or DARPP-32 shRNAs were injected into the left thorax of SCID mice and imaged for luminescence on indicative days. **d** Luciferase-labeled human A549 cells overexpressing control, DARPP-32 or t-DARPP clones were orthotopically injected into the left thorax of SCID mice and imaged for luminescence on indicative days. Representative in vivo images of SCID mice are depicted. Total luminescence intensity (photon count) was calculated using molecular imaging software. The colored bar represents the numerical value of luminescence. Error bars indicate SEM. *P < 0.05, ***P < 0.001 and ****P < 0.0001, one-way ANOVA followed by Dunnett’s test for multiple comparison

Based on our findings that DARPP-32 promotes lung cancer survival and migration, combined with previous studies implicating DARPP-32 as an oncogenic factor contributing to breast cancer and gastric tumor progression^12,46^, we sought to determine whether DARPP-32 drives lung cancer growth in vivo. To this end, we tested whether DARPP-32 ablation reduces lung tumor growth in an orthotopic xenograft mouse model. Briefly, we injected luciferase-labeled human A549 NSCLC cells into the left thorax of anesthetized SCID (severe combined immunodeficient) mice, allowed establishment of the lung tumor and then xenogen imaged the mice regularly over the course of 5 to 6 weeks. We observed a substantial decrease in lung tumor growth in mice challenged with DARPP-32 ablated A549 cells compared to mice challenged with cells transduced with control LacZ shRNA (Fig. 6a). Correspondingly, we found decreased tumor growth in mice orthotopically injected with DARPP-32 ablated H1650 (Fig. 6b) or H226 (Fig. 6c) human lung cancer cells. These findings support our hypothesis that DARPP-32 depletion inhibits human lung tumor growth. We next sought to determine whether overexpression of DARPP-32 promotes lung tumor growth in vivo. To address this question, we injected luciferase-labeled human A549 NSCLC cells stably overexpressing exogenous DARPP-32 or t-DARPP into the left thorax of anesthetized SCID mice. We demonstrated that DARPP-32 and t-DARPP overexpression promotes lung tumor growth in mice (Fig. 6d). Taken together, our data suggest DARPP-32 proteins drive lung tumorigenesis and inhibition of DARPP-32 reduces lung cancer growth.

### Elevated t-DARPP and tumor (T) staging score correlate in NSCLC patients

**Fig. 7:**
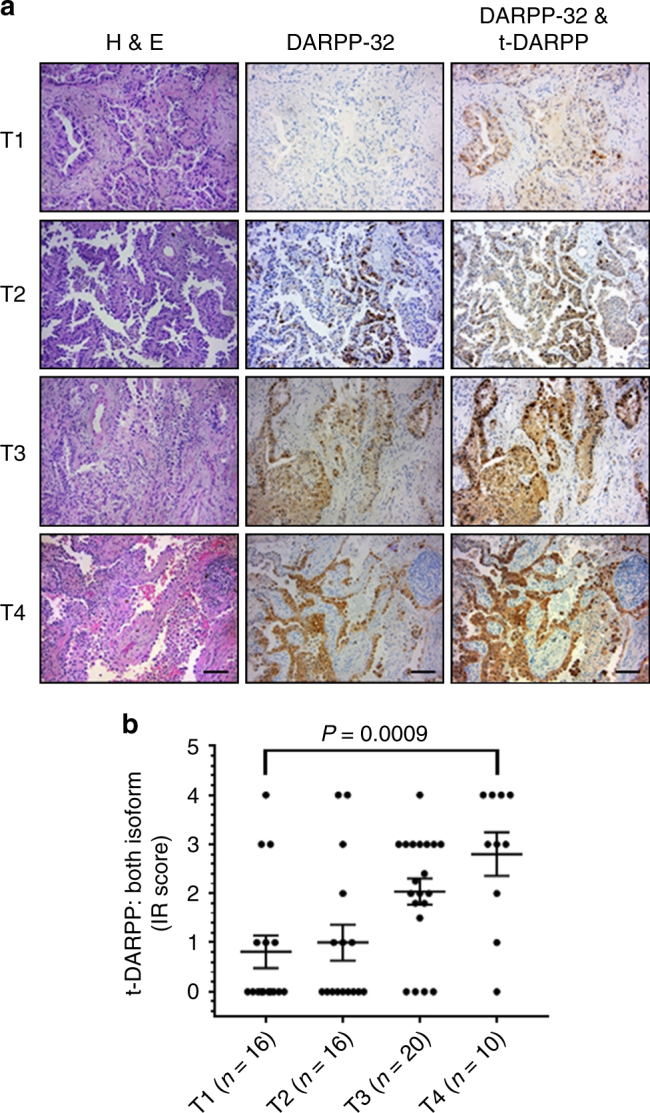
Elevated t-DARPP protein expression positively correlates with tumor (T) staging score in lung adenocarcinoma patients. **a** Immunohistochemistry was performed on human NSCLC serially sectioned specimens using an N-terminal DARPP-32 antibody to exclusively detect DARPP-32 and a C-terminal DARPP-32 antibody to detect both DARPP-32 and t-DARPP. Scale bar, 50 µm. **b** Differential immunohistochemistry was used to quantify t-DARPP protein expression in 62 human lung cancer tissue samples. Each tissue was scored based on the percentage of tumor cells stained positive multiplied by the staining intensity (i.e., 0 = none, 1 = weak, 2 = moderate, 3 = strong expression) to yield an immune reactive (IR) score. The IR score for t-DARPP protein expression was calculated by subtracting the IR score of DARPP-32 (detected with N-terminal antibody) from the IR score of both DARPP-32 isoforms (detected with C-terminal antibody). Each point on the graph represents an individual tissue. Error bars indicate SEM. The *P* value has been calculated using one-way ANOVA followed by Dunnett’s test for multiple comparison

We aimed to elucidate the clinical relevance of DARPP-32 given its role in promoting tumor growth in mouse models of human NSCLC. Correspondingly, previous studies have linked upregulation of DARPP-32 and t-DARPP with breast, gastric and colorectal cancer^9,14,36,46–48^. To assess DARPP-32 and t-DARPP expression in NSCLC patients, we obtained tissue specimens from 62 lung adenocarcinoma patients and performed previously described differential immunohistochemistry to detect the expression of DARPP-32 and t-DARPP^12^. Specifically, we individually immunostained serial whole tissue sections of formalin-fixed, paraffin-embedded tissue blocks corresponding to each patient with two distinct DARPP-32 antibodies that: (1) detects both DARPP-32 and t-DARPP via a C-terminal epitope present in both isoforms, or (2) exclusively detects DARPP-32 through an N-terminal epitope absent in the N-terminally truncated t-DARPP isoform (antibody specificity controls are shown in Supplementary Fig. 11a, b). Because most of the patients in our cohort had stage III lung adenocarcinoma (Supplementary Table 1), we used the tumor (T) staging score (i.e., from the 7th edition of the lung cancer TNM (tumor, node, metastasis) staging system^49^), which represents the size of the primary tumor and whether it has grown into nearby areas, as a metric of tumor progression and growth. A pulmonary pathologist (A.C.R.) scored the percentage of positive tumor cells and their staining intensity of DARPP-32 only and both isoforms (DARPP-32 and t-DARPP) using a scale of 0–3 (i.e., 0 = none, 1 = weak, 2 = moderate, 3 = strong expression). Using the resulting pathological scoring, we calculated an immune reactive (IR) score for each specimen based on the percentage of tumor cells staining positive and the staining intensity in those cells (IR score = percentage of tumor cells×staining intensity). We found that high relative expression of t-DARPP correlates with worsening T staging score in the 62 lung adenocarcinoma specimens examined by immunohistochemistry (Fig. 7a, b). Our results suggest that a subset of patients with advanced lung adenocarcinoma exhibit elevated levels of t-DARPP protein and that upregulation of t-DARPP appears to be associated with T staging score.

### t-DARPP, NF-ĸB2 and IKKα upregulation is linked to decreased NSCLC patient survival

**Fig. 8:**
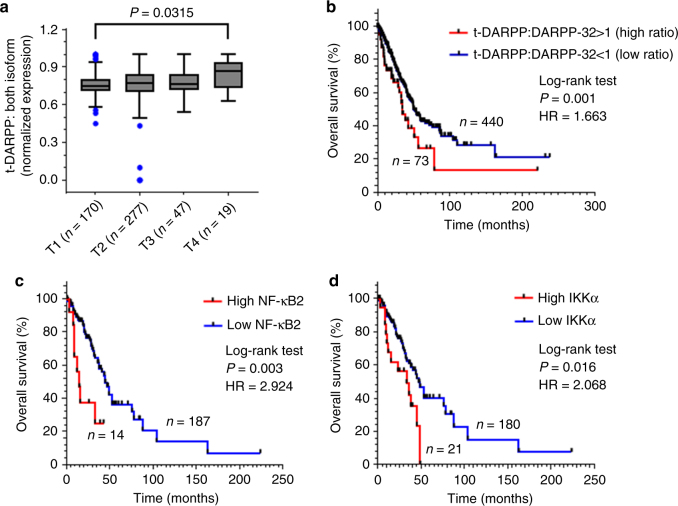
Elevated t-DARPP, NF-ĸB2 and IKKα transcripts correlate with decreased lung adenocarcinoma patient survival. **a** Quantification of t-DARPP mRNA expression in 513 human lung cancer tissue samples. Blue dots indicate outliers. T1–T4 represents tumor (T) staging scores from TNM system. **b** Kaplan–Meier plot showing overall survival within the total cohort of 513 NSCLC patients based on the expression of t-DARPP and DARPP-32 mRNAs. **c** Kaplan–Meier curve depicting overall survival within the total cohort of 201 NSCLC patients based on the expression of NF-ĸB2 or **d** IKKα mRNAs. The normalized read count for DARPP-32, t-DARPP, NF-ĸB2 and IKKα mRNAs were obtained from The Cancer Genome Atlas dataset (TCGA). The difference between the two groups was calculated using log-rank (Mantel–Cox) test. HR hazard ratio

We utilized a bioinformatics approach to validate our finding that high relative t-DARPP expression correlates with tumor growth in lung adenocarcinoma patients. We assessed relative DARPP-32 and t-DARPP transcript expression in specimens corresponding to 513 human lung adenocarcinoma patients cataloged in The Cancer Genome Atlas (TCGA). Interestingly, we found that expression of t-DARPP increases with advancing tumor (T) stages in lung adenocarcinoma (Fig. 8a). As assessed by Kaplan–Meier survival curve, we observed that patients with high t-DARPP to DARPP-32 ratio showed substantially decreased survival relative to lung adenocarcinoma patients with low t-DARPP expression (Fig. 8b). On the contrary, there is no substantial difference in the survival outcome of lung adenocarcinoma patients when we consider only total t-DARPP levels independently of relative DARPP-32 expression (Supplementary Fig. 12). Our findings indicate that t-DARPP expression is an important determinant of survival in lung adenocarcinoma patients.

Given our findings that DARPP-32 isoforms regulate non-canonical NF-ĸB2-mediated cell migration, we asked whether expression of NF-ĸB2 or IKKα is associated with overall survival of lung adenocarcinoma patients. RNA-sequencing (RNA-Seq) expression data from 201 human lung adenocarcinoma tissue samples was used to generate Kaplan–Meier survival curves. Our results reveal decreased survival in the patients with high expression of NF-ĸB2 and IKKα transcripts compared to low expressers of those mRNAs (Fig. 8c, d). Thus, upregulation of NF-ĸB2 and IKKα expression is associated with decreased overall patient survival and may predict poor clinical outcome in lung adenocarcinoma patients.

## Discussion

For the first time to our knowledge, we demonstrate that DARPP-32 and its splice variant t-DARPP stimulate lung cancer cell survival and migration to promote oncogenesis, and we show that elevated t-DARPP isoform levels in NSCLC patients are associated with increased tumor staging and worsened patient survival. The role of DARPP-32 and t-DARPP in cancer has emerged beyond their classical function as modulators of dopamine-mediated neurotransmission, highlighting their importance in the regulation of physiological and pathological effects. For example, alternations in expression of DARPP-32 and t-DARPP have been implicated in schizophrenia, bipolar disorder and Alzheimer’s disease^50,51^ as well as numerous types of tumors, including breast, gastric, prostate, esophageal and colon cancers^9,12,15,52^. Since investigation of the frequent amplification at the 17q12 locus in gastric cancers implicated DARPP-32 and t-DARPP in oncogenesis^9,10^, numerous studies have demonstrated the role of these proteins in cancer cell survival, drug resistance, migration, invasion and angiogenesis^17^.

Our results suggest DARPP-32 and t-DARPP promote NSCLC cell survival through activation of Akt and Erk1/2 signaling by protecting cells from apoptotic cell death (Figs. 1 and 2 and Supplementary Fig. 1). Correspondingly, overexpression of DARPP-32 and t-DARPP in human gastrointestinal adenocarcinoma cells was shown to cause a fourfold reduction in apoptosis^10^. The T75 phosphorylation residue shared by DARPP-32 and t-DARPP was attributed to promoting cell survival^10^, and a follow-up report by the same group suggested increased activation of Akt and Bcl2 is mechanistically responsible for t-DARPP-mediated cancer cell survival^18^. Evasion of apoptosis is a major underlying mechanism in the ability of cancer cells to acquire resistance to molecular targeted therapies^53^. El-Rifai and colleagues^19^ demonstrated that DARPP-32 promotes cell survival and gefitinib resistance in gastric cancer cells by stimulating EGFR phosphorylation and activating phosphatidylinositol 3-kinase/Akt signaling. DARPP-32 stimulated resistance to pro-apoptotic proteins through induction of pro-survival molecule Bcl-xL through Src/STAT3 (signal transducer and activator of transcription 3) signaling cascades^16^. Numerous reports have implicated t-DARPP in breast cancer patients acquiring resistance to trastuzumab (Herceptin), a monoclonal antibody targeting the ERBB2 (Her2/neu) receptor. Collectively, these studies demonstrated that t-DARPP drives breast cancer cell resistance to trastuzumab through inhibition of apoptotic caspase-3 and activation of pro-survival Akt signaling through its T75 residue, common among both DARPP-32 isoforms^13,52,54^. Another report showed t-DARPP promotes trastuzumab resistance in esophageal adenocarcinoma cells through similar mechanisms^48^. In both breast and esophageal cancer, t-DARPP physically interacted with ERBB2 in a protein complex to mediate trastuzumab resistance^48,54^. Like previous studies in gastric, breast and esophageal cancers, our studies suggest DARPP-32 and t-DARPP promote cell survival through upregulation of Akt signaling. Despite this observation and the common underlying mechanistic evidence, future studies beyond the scope of this study are necessary to determine whether t-DARPP promotes resistance to specific molecular targeted NSCLC therapies.

Like pro-survival mechanisms, increased cell migration also contributes to cancer cell growth and resistance to molecular targeted therapies. Given the well-established association between DARPP-32 isoforms and acquired drug resistance in cancer, it is unsurprising that several reports and detailed reviews have described the role of DARPP-32 in breast and gastric cancer cell migration and invasion^17,40,55^. Correspondingly, we provide evidence that DARPP-32 and t-DARPP promote NSCLC cell migration based on in vitro scratch (Fig. 3) and spot assays (Supplementary Figs. 3 and 4). In line with our observations, other studies have demonstrated that overexpression of DARPP-32 promotes tumor cell invasion in gastric and colorectal cancers^40,47^. Conversely, DARPP-32 has been shown to inhibit breast cancer cell migration through a dopamine D1 receptor-dependent mechanism^55^. A subsequent in vitro study has revealed that PP-1 inhibition regulated by phosphorylation of DARPP-32 at residue T34 is critical for modulating cell migration in breast cancer^39^. Taken together, the regulation of cancer cell migration by DARPP-32 is likely cell and tumor type dependent.

We identify a physical interaction between DARPP-32 and IKKα that suggests DARPP-32 regulates non-canonical NF-κB2 signaling to control NSCLC migration (Fig. 4 and Supplementary Figs. 5, 6 and 7). Knockdown of IKKα, as well as independently silencing NF-κB2, decreased migration of human lung adenocarcinoma cells (Fig. 5a, b). Based on our findings, we propose that DARPP-32 activates IKKα through an unknown NIK-independent mechanism that leads to IKKα-mediated phosphorylation of NF-ĸB2 p100, ubiquitination and partial degradation of p100 to p52, and translocation of NF-ĸB2 p52 to the nucleus where it acts as a transcription factor to modulate expression of genes involved in cell migration (Supplementary Fig. 10). A recent report has demonstrated that *Helicobacter pylori* infection induces canonical NF-ĸB1-mediated transcriptional upregulation of DARPP-32 mRNA and protein, which counteracts *H. pylori*-mediated cell death through activation of Akt^35^. Therefore, we investigated whether non-canonical NF-κB2 signaling altered DARPP-32 protein expression, but observed no effect (Supplementary Fig. 8). While canonical NF-ĸB1 pathway activation has been linked to the growth and survival of many solid and hematological malignancies, the role of non-canonical NF-ĸB2 signaling in cancer is still emerging^22,42^. However, studies suggest the NF-ĸB2 pathway is activated in cancer through viral oncogenes, mutations in pathway components and upregulation of upstream components of the pathway^42^, the latter of which is supported by our results, suggesting DARPP-32 promotes activation of non-canonical NF-ĸB2 signaling in lung cancer through an interaction with IKKα.

We demonstrate that stable overexpression of DARPP-32 and t-DARPP in human NSCLC cells orthotopically implanted into the thoracic cavity of SCID mice promotes tumor growth (Fig. 6d). Correspondingly, mice that received an orthotopic xenograft of shRNA-mediated DARPP-32-silenced NSCLC cells exhibited decreased tumor growth relative to controls (Fig. 6a–c). El-Rifai and colleagues^48^ have shown overexpression of t-DARPP in human OE19 esophageal adenocarcinoma subcutaneously xenografted into athymic nude mice stimulates tumor growth. Using a similar xenograft mouse model, they have subsequently demonstrated shRNA-mediated knockdown of DARPP-32 reduces gastric tumorigenesis^19,56^ and overexpression of DARPP-32 in AGS human gastric adenocarcinoma cells promotes in vivo tumor growth^46^. To the best of our knowledge, our study is the first to assess DARPP-32 knockdown as well as DARPP-32 and t-DARPP overexpression in an orthotopic cancer xenograft mouse model. Importantly, our in vivo results showing DARPP-32 and t-DARPP promote NSCLC oncogenesis coincide with similar findings in esophageal and gastric cancer subcutaneous xenograft models.

Based on differential immunostaining of over 60 human NSCLC specimens, we describe that high relative expression of t-DARPP correlates with tumor staging in lung adenocarcinoma patients (Fig. 7). Similar differential immunohistochemistry approaches in serial tissue sections have been previously used to distinguish between detection of DARPP-32 only (N-terminal antibody) versus both isoforms (C-terminal antibody). Two independent studies have demonstrated a subset of primary human breast cancer specimens exhibit elevated t-DARPP protein levels relative to DARPP-32^12,52^. Using a genetic spontaneous murine model of breast cancer, Christenson and Kane^36^ have found DARPP-32 was expressed in normal mammary tissue and in some breast tumors, whereas t-DARPP was detected exclusively in tumors, typically at higher or equal levels as DARPP-32. This transition from DARPP-32 to t-DARPP observed during breast tumorigenesis corresponds to our pathological and bioinformatics findings linking upregulation of t-DARPP expression with increased NSCLC growth and worsened patient survival. The DARPP-32 to t-DARPP isoform shift in cancer may be directed by the SRp20 splicing factor, which has been shown to physically associate with DARPP-32^56^. The upregulation of t-DARPP in NSCLC progression suggests its expression stimulates oncogenesis. Thus, t-DARPP may represent a promising molecular target in NSCLC as well as possess prognostic value.

## Methods

### Cell culture

Human NSCLC cell lines A549, H1650 and H226 as well as the transformed human embryonic kidney epithelial cell line, HEK-293T, were purchased from American Type Culture Collection and maintained according to the manufacturer’s instructions. HEK-293T cells were cultured in Dulbecco’s modified Eagle’s medium (Corning) and lung cancer cell lines were cultured in RPMI-1640 medium (Corning). Media were supplemented with 10% fetal bovine serum (Millipore) and 1% Penicillin/Streptomycin antibiotics (Corning). All cell lines were certified by the indicated cell bank and routinely authenticated by morphologic inspection.

### Transient transfections

The 5 × 10^5^ A549 or H1650 cells were seeded in 60-mm cell culture plates and incubated for 24 h in RPMI-1640 medium. Cells were then washed with phosphate-buffered saline (PBS), suspended in OPTI-MEM reduced serum medium (Gibco) and transfected with 2 µg of pMMP-LacZ or pMMP-DARPP-32 plasmids using Polyfect transfection reagent (Qiagen) according to instructions from the manufacturer. After 4 h, antibiotic-containing complete RPMI-1640 medium was added and cells were grown until they had established a confluent monolayer.

### Generation of stable cell lines

Expression constructs of human DARPP-32, t-DARPP and DARPP-32 T34A complementary DNA in pcDNA3.1 were a generous gift from Dr. Wael El-Rifai at the Vanderbilt University Medical Center^46^. The Flag-tagged coding sequence of DARPP-32, t-DARPP and T34A DARPP-32 were subcloned into the retroviral (pMMP) vector. The pMMP plasmid and its corresponding pMMP-LacZ control construct were kindly provided by Dr. Debabrata Mukhopadhyay at Mayo Clinic in Jacksonville, Florida^57^. Retrovirus was produced by transfecting HEK-293T cells with pMMP vectors encoding the target genes. The retrovirus was isolated 48 h after transfection and used immediately to transduce A549, H1650 and H226 lung cancer cell lines as previously described^57^.

Four to five different lentiviral shRNA pLKO.1 constructs (Sigma-Aldrich) were used to silence protein expression of each target, including DARPP-32, NF-ĸB2 and IKKα. pLKO.1-LacZ shRNA (Sigma-Aldrich) was used as a corresponding control. To prepare the lentivirus, shRNA pLKO.1 constructs along with their corresponding packaging plasmids were tranfected in HEK-293T cells. At 48 h after transfection, the lentivirus was collected and used immediately for transduction of A549, H1650 and H226 lung cancer cell lines. Stable knockdown cells were used for experiments after 72 h of puromycin (Sigma) selection^58^.

### Cell survival assay

A549, H226 and H1650 human NSCLC cell lines were each plated in a 96-well microplate at a concentration of 3000 cells per well. Cell viability was assessed after 72 h of incubation using CellTiter 96^®^ AQueous One System (Promega). Absorbance was recorded at 490 nm using an Epoch microplate spectrophotometer (Biotek). The average of three independent experiments has been reported.

### Cell proliferation analysis by BrdU labeling

Human NSCLC cells were seeded at a density of 1 × 10^5^ cells per 60 mm plate. The following day, BrdU (30 µM; Sigma-Aldrich) diluted in fresh medium was administered to the cells for 30 min. The cells were harvested, fixed and processed for incubation with primary mouse anti-BrdU monoclonal antibody (Roche) and subsequently secondary allophycocyanin (APC)-conjugated goat anti-mouse antibody (Biotium). BrdU-positive cells were counted. Finally, the cells were stained with propidium iodide (Sigma-Aldrich) for flow cytometry analysis. The average of three separate experiments has been documented.

### Apoptosis analysis

The 1 × 10^5^ LacZ control- or DARPP-32 shRNA-transduced A549, H1650 and H226 cells were plated in 60 mm dishes. Cells were then harvested, washed with PBS and stained with annexin V-APC (BD Biosciences) following 24 h of incubation. Additional exposure to propidium iodide (BD Biosciences) made it possible to differentiate early apoptotic cells (annexin-positive and propidium iodide-negative) from late apoptotic cells (annexin-positive and propidium iodide-positive). Apoptotic cell death was assessed by counting the number of cells that stained positive for annexin V-APC. The average of three independent experiments has been reported.

### Antibodies

Antibodies (200 µg ml^–1^) against DARPP-32 (cat no.: sc-11365; dilution 1:100) and α-Tubulin (cat no.: sc-5286; dilution 1:500) were purchased from Santa Cruz Biotechnology. Antibodies (1 µg/µl) against PARP (cat no.: 9542; dilution 1:1000), caspase-3 (cat no.: 9662; dilution 1:1000), cleaved caspase-3 (cat no.: 9664; dilution 1:1000), phosphorylated Akt (S473; cat no.: 4060; dilution 1:1000), total Akt (cat no.: 4691; dilution 1:1000), phosphorylated p44/42 MAPK (T202/Y204; cat no.: 4370; dilution 1:1000), total p44/42 MAPK (cat no.: 4695; dilution 1:1000), phosphorylated NF-κB2 p100 (S866/870; cat no.: 4810; dilution 1:500), total NF-κB2 p100/52 (cat no.: 4882; dilution 1:1000), phosphorylated IKKα/β (S176/180; cat no.: 2697; dilution 1:500), total IKKα (cat no.: 11930; dilution 1:1000), histone H3 (cat no.: 4499; dilution 1:1000) and FLAG tag (cat no.: 14793; dilution 1:2000) were obtained from Cell Signaling Technology. An antibody that exclusively detects DARPP-32 (cat no.: ab40801; dilution 1:1300) via an N-terminal epitope absent in t-DARPP was purchased from Abcam and used for immunohistochemistry. Horseradish peroxidase-conjugated anti-rabbit (cat no.: 7074; dilution 1:5000) and anti-mouse (cat no.: 7076; dilution 1:5000) secondary antibodies (1 µg µl^–1^) were also purchased from Cell Signaling Technology. Full scans of all western blots represented in main figures or supplementary figures are shown in Supplementary Figs. 13–23.

### Nuclear extract preparation

The 5 × 10^6^ human NSCLC cells were suspended in hypotonic buffer (20 mM Tris-Cl pH 7.4, 10 mM NaCl, 3 mM MgCl_2_, protease inhibitor cocktail and 1 mM phenylmethylsulfonyl fluoride (PMSF) Cell Signaling Technology) and incubated on ice for 15 min. Nonionic detergent NP-40 (10%; Sigma-Aldrich) was then added to the cell suspension, which was mixed vigorously. Next, the cell homogenate was centrifuged at 5000 rpm for 10 min at 4 °C. The supernatant was collected as the cytoplasmic fraction, and the pellet was suspended in cell extraction buffer (Thermo Fisher Scientific) supplemented with protease inhibitor cocktail (Roche) and 1 mM PMSF. The suspension was incubated on ice for 30 min with intermittent vortexing. Finally, the sample was centrifuged at 14,000 × *g* for 30 min at 4 °C, and the supernatant was collected as nuclear extract.

### Immunoblotting

A549, H1650 and H226 cells were sonicated and lysed in RIPA buffer (Millipore) containing protease inhibitor cocktail (Roche). Proteins were separated via 4–20% gradient sodium dodecyl sulfate–polyacrylamide gel electrophoresis (Bio-Rad) and transferred to polyvinyl difluoride membranes (Millipore). Membranes were blocked with 5% bovine serum albumin (Sigma-Aldrich) and incubated with primary and secondary antibodies. Antibody-reactive protein bands were detected by enzyme-linked chemiluminescence (Thermo Fisher Scientific).

### Immunoprecipitation

Human lung cancer cells were homogenized and lysed in RIPA buffer (Millipore) supplemented with protease inhibitor cocktail (Roche). Protein concentration was measured using the Quick Start Bradford protein assay (Bio-Rad) and 500 µg of protein lysate was loaded into the supplied spin column (Catch and Release Immunoprecipitation Kit; Millipore). Immunoprecipitation was achieved by following the manufacturer’s protocol (cat no.: 17–500; Millipore).

### Immunofluorescence

A549, H1650 and H226 cells were fixed in 4% paraformaldehyde (Boston Bioproducts), permeabilized in cold methanol (Fisher) and immunofluorescence staining was performed using an antibody against NF-κB2 p100/52 (Cell Signaling Technology; cat no.: 3017). The cells were then washed with PBS and incubated with Alexa Fluor 568-conjugated anti-rabbit secondary antibody (Molecular Probes; cat no.: A11036). All images were captured by confocal microscopy (Nikon; 60× objective, 1.27 NA) and processed using ImageJ software (Version 1.6.0_24; https://imagej.nih.gov/ij). The 4',6-diamidino-2-phenylindole (DAPI)-stained nuclei were segmented manually by drawing a circular region of interest. Nuclear colocalization of NF-κB2 p52 (red fluorescent signal) with DAPI was measured. The mean nuclear fluorescence was calculated and plotted in GraphPad Prism software (Version 7).

### Quantitative real-time PCR (qRT-PCR)

Total RNA was isolated from A549 and H1650 cell lines using an RNeasy Plus kit (Qiagen) according to the manufacturer’s instructions. Isolated RNA (100 ng) was subjected to qRT-PCR analysis using iTaq^™^ Universal SYBR^®^ Green One-Step Kit (Bio-Rad) performing using the 7500 Real-Time PCR System (Applied Biosystems). The comparative threshold cycle method (ΔΔC_t_) was used to quantify relative amounts of transcripts with *B2M* (β-2-Microglobulin) as an endogenous reference control. Primer sequences (forward and reverse, respectively) used for qRT-PCR were as follows. *EZH2*: 5ʹ-GGGACAGTAAAAATGTGTCCTGC-3ʹ and 5‘-TGCCAGCAATAGATGCTTTTTG-3ʹ; *BIRC3*: 5ʹ-GCCCTCTAGTGTTCTAGTTAATCC-3ʹ and 5ʹ-TACTCACACCTTGGAAACCAC-3ʹ; *B2M*: 5ʹ-TCTCTGCTGGATGACGTGAG-3ʹ and 5ʹ-TAGCTGTGCTCGCGCTACT-3ʹ.

### Immunohistochemistry

Human lung adenocarcinoma tissue specimens were obtained from 62 NSCLC patients at Mayo Clinic in Rochester, MN, in accordance with institutional review board-approved protocols. We performed previously described differential immunohistochemistry^12^ using an N-terminal antibody that exclusively recognizes DARPP-32 (Abcam; cat no.: ab40801; dilution 1:1300). We used another C-terminal antibody that recognizes both DARPP-32 and t-DARPP (Santa Cruz Biotechnology; cat no.: sc-11365; 1:100). Formalin-fixed, paraffin-embedded whole tissues were serially sectioned and immunostained for DARPP-32 using a Bond Autostainer (Leica) as previously described^21^. Hematoxylin and eosin staining was also performed. In each lung tumor specimen, the intensity and prevalence of DARPP-32 staining in various cell types was scored by a pulmonary pathologist (A.C.R.).

### Scratch wound assay

A549 and H1650 cells were seeded in 60 mm culture dishes at an appropriate density to achieve a confluent monolayer. After 16 h, a linear scratch wound was generated using a sterile 20 µl pipette tip. Cells were imaged at time 0 and 14 h post scratch induction. All the images were captured using a 4× Plan S-Apo 0.16 NA objective on an EVOS FL cell imaging system (Thermo Fisher Scientific). The images were analyzed using ImageJ software and cell migration was quantified as previously described^59^.

### Spot assay

A549 and H1650 human lung cancer cells were trypsinized and suspended in RPMI-1640 medium (Corning) at a concentration of 5 × 10^4^ cells per µl. Cells (2.5 × 10^5^ in 5 µl) were then mixed with Matrigel® Basement Membrane Matrix (Corning) in a 1:1 ratio and pipetted as a spot in a 60 mm culture dish. Matrigel containing cell suspension (i.e., the spot) was allowed to solidify by incubating at 37 °C for 5 min. Thereafter, medium was added and images were captured using a 4× Plan S-Apo 0.16 NA objective on an EVOS FL cell imaging system (Thermo Fisher Scientific). After 96 h of incubation, the spots were imaged again and cell migration was calculated as previously described^60^.

### In vivo orthotopic lung cancer model

The 6–8-week-old pathogen-free SCID/NCr mice were purchased from the Charles River Laboratories. Mice were allowed 1 week to acclimate to their surroundings, bred, maintained under specific pathogen-free conditions in a temperature-controlled room with alternating 12 h light/dark cycles and fed a standard diet. The 8–12-week-old male and female mice were orthotopically injected with 1 × 10^6^ luciferase-labeled human A549, H226 and H1650 lung cancer cells suspended in 80 μl PBS and Matrigel. After establishment of the lung tumor, mice were imaged using an In-Vivo Xtreme xenogen imaging system (Bruker) to measure luciferase intensity. To determine tumor growth, luciferase intensity was calculated using Bruker molecular imaging software and plotted over time in GraphPad Prism 7 software. All animal studies were performed in accordance with the protocols approved by the University of Minnesota Institutional Animal Care and Use Committee.

### RNA-Seq analysis

The RNA-Seq isoform expression data of human lung adenocarcinoma tissue specimens in TCGA were used for correlation analysis with the clinical variables. The clinical information of 513 patients were downloaded from the Xena Public Data Hubs (https://xena.ucsc.edu). The processed RNA-Seq data (version 2 Level 3) for the normalized isoform expression of the 513 tumor samples were downloaded from the Genomic Data Commons Legacy Archive (https://gdc-portal.nci.nih.gov/legacy-archive). DARPP-32 contains five isoforms in the downloaded data. Among the five isoforms, uc002hrz.2, uc002hsa.2 and uc010cvx.2 are longer isoforms (i.e., representing full length DARPP-32) starting at the original start codon, while uc002hsb.2 and uc002hsc.2 are shorter isoforms (i.e., representing alternate isoform t-DARPP) sharing another downstream start codon. The log2(*x* + 1) transformed FPKM (fragments per kilobase of transcript per million mapped reads) value normalized by RSEM (reads per kilobase of transcript per million mapped reads) for isoform and gene expressions were used in further application. Based on the t-DARPP expression, the Kaplan–Meier survival graph was created by using GraphPad Prism 7 software.

The NF-ĸB2 and IKKα expression data and the clinical variables of 201 human lung adenocarcinoma tissue specimens were obtained from cBioPortal for Cancer Genomics (http://cbioportal.org)^61,62^. Patients were categorized into two separate groups based on the mRNA expressions (normalized read count) and Kaplan–Meier survival curve was generated by using GraphPad Prism 7 software.

### Statistics

Statistical comparisons were performed with one-way analysis of variance (ANOVA) and values of *P* < 0.05 were considered significant. For multiple comparison between groups, Dunnett test was performed after one-way ANOVA in all pertinent experiments. In survival curve analysis of TCGA data, we utilized log-rank test to compare groups. Data are expressed as mean ± SEM and representative of at least three independent experiments.

### Data availability

The authors declare that the data supporting the findings of this study are available within the article and its supplementary information.

## Electronic supplementary material

Supplementary Information
