# Supplementary material for: DARPP-32 and t-DARPP promote non-small cell lung cancer growth through regulation of IKKα-dependent cell migration
Source: Commun Biol. 2018 May 3;1:43. doi: 10.1038/s42003-018-0050-6 (PMC5959014; doi:10.1038/s42003-018-0050-6)
Supplement: Supplementary file 1 — Supplementary Information [file 42003_2018_50_MOESM1_ESM.pdf]

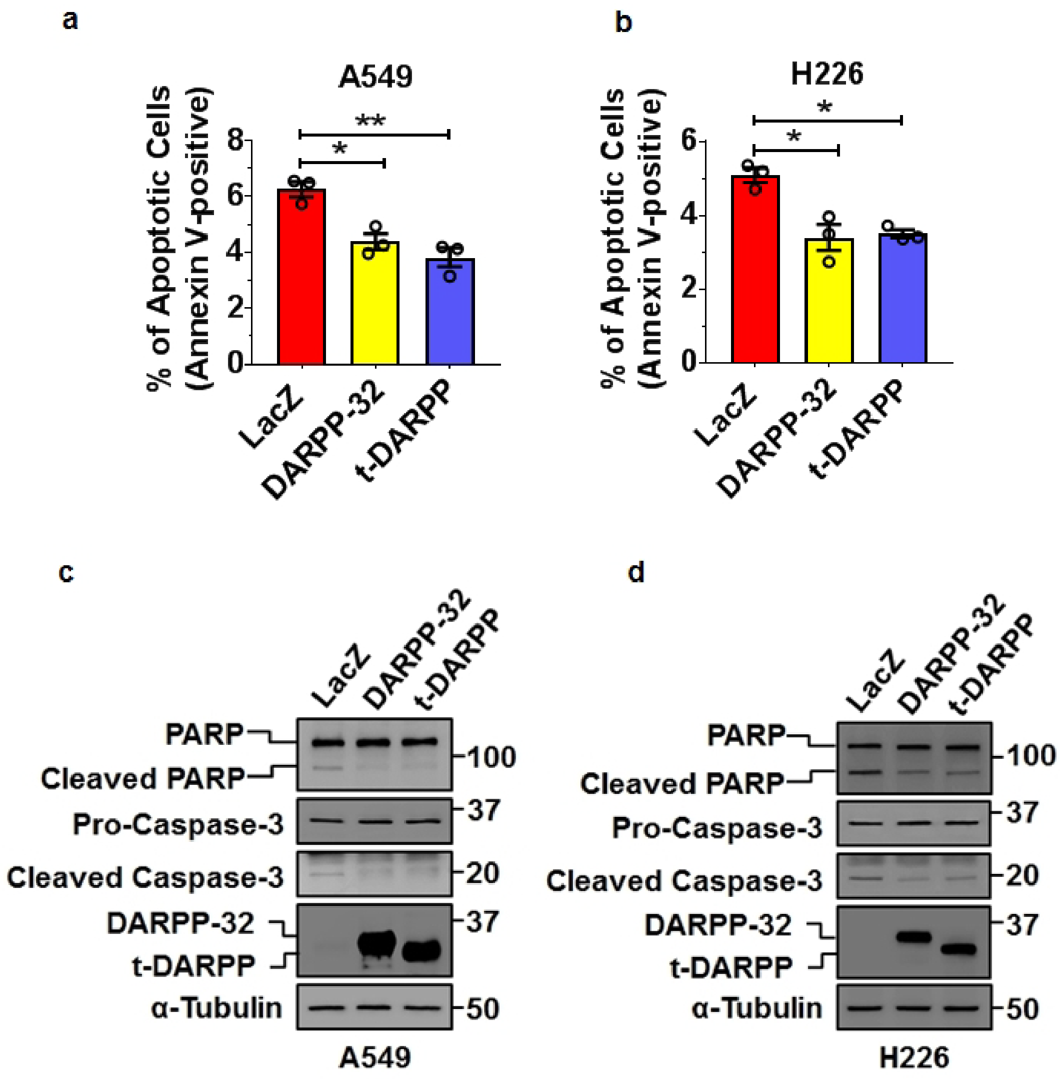

Supplementary Figure 1: DARPP-32 inhibits apoptosis. **a** A549 and **b** H226 cells overexpressing DARPP-32 isoforms were seeded into 60-mm culture dishes for 16h. Flow cytometry-based apoptosis assays were performed following incubation with anti-annexin V antibodies conjugated with APC. **c** DARPP-32 overexpressing A549 and **d** H226 cells were subjected to immunoblot analysis using antibodies that detect cleaved and uncleaved PARP, cleaved and uncleaved (i.e. pro-) Caspase-3, DARPP-32 and α-tubulin (loading control). Uncropped images of depicted immunoblots are shown in Supplementary Fig. 20a and 20b. All bar graphs represent mean ± SE of at least 3 independent experiments. Each open circle on a graph represents an independent experiment. **P*<0.05 and ***P*<0.01, one-way ANOVA followed by Dunnett's test for multiple comparison.

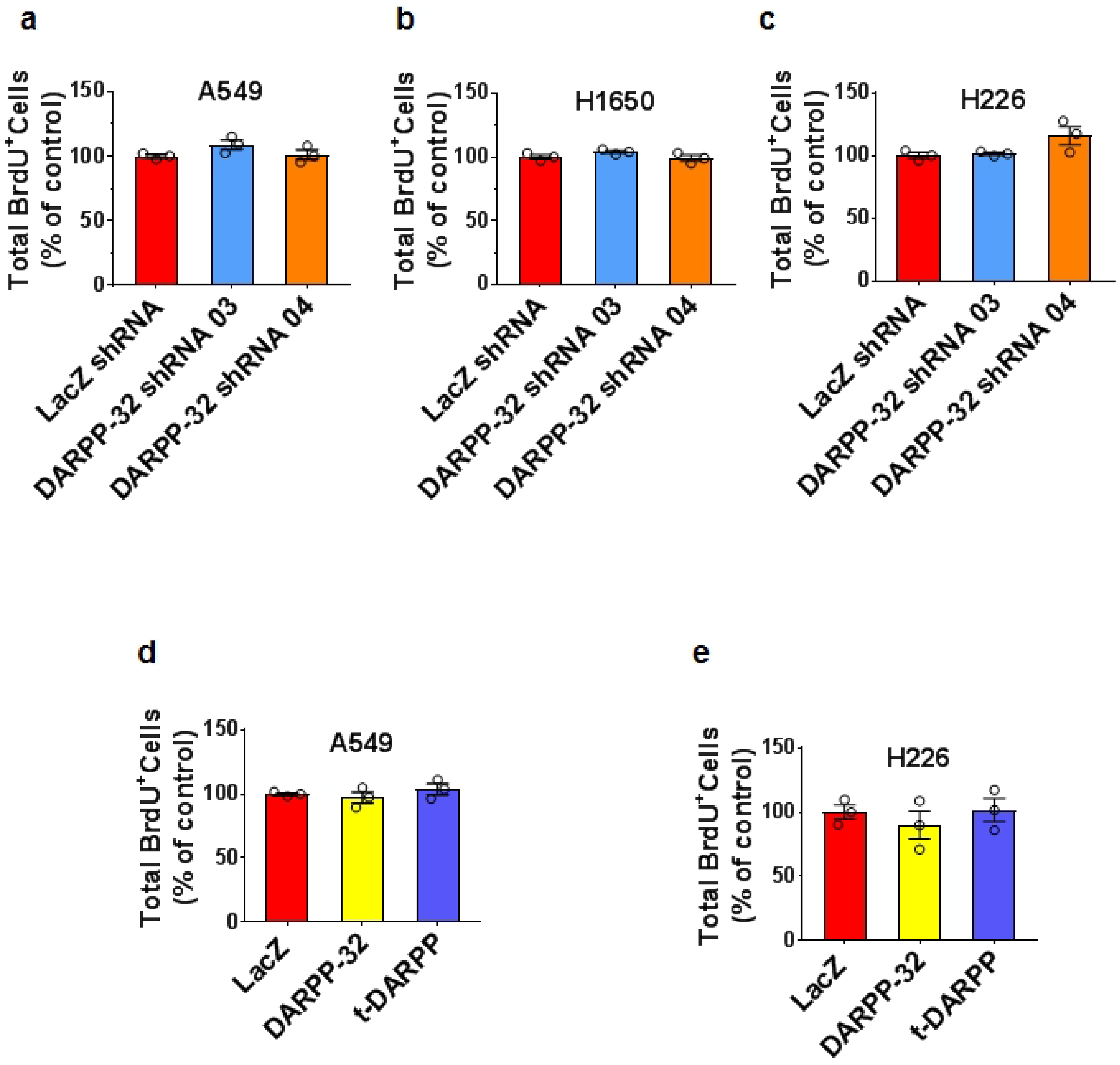

Supplementary Figure 2: DARPP-32 does not regulate cell cycle progression. **a** A549, **b** H1650 and **c** H226 cells transduced with control or DARPP-32 shRNAs were seeded into 60-mm culture dishes for 16h. Flow cytometer-based BrdU cell proliferation assays were performed following incubation with anti-BrdU antibodies conjugated with APC. **d** A549 and **e** H226 cells were transduced with retrovirus containing control (LacZ), DARPP-32 or t-DARPP overexpressing clones. Flow cytometry-based BrdU cell proliferation assays were conducted and total BrdU$^+$ cells were calculated. All bar graphs represent mean ± SE of at least 3 independent experiments.

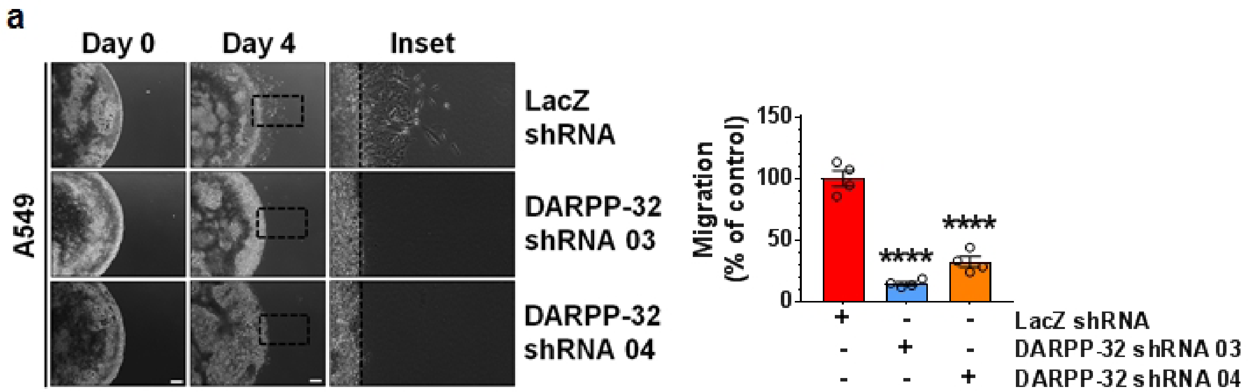

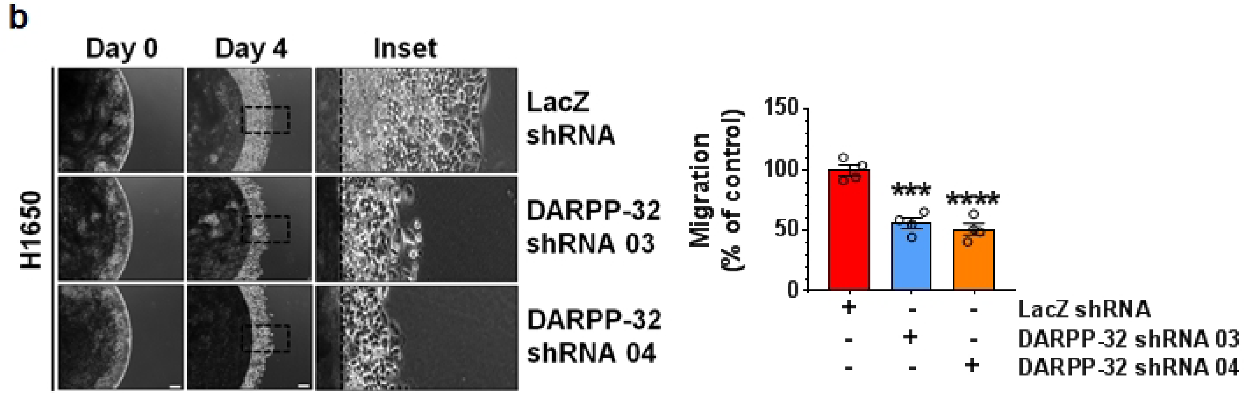

Supplementary Figure 3: Knockdown of DARPP-32 reduces cell migration by spot assay. **a** A549 and **b** H1650 cells transduced with lentivirus encoding control or DARPP-32 shRNAs were mixed with Matrigel and spotted. Representative images depict one half of the spot at 0 and 4 days post-plating. Enlarged insets of day 4 images are depicted. The dashed line indicates the edge of the spot at day 0. Experiments were repeated at least three times. Scale bar, 200 µm. Distance travelled by the migratory cells were calculated using ImageJ software. Results represent mean ± SE. ****P*<0.001 and *****P*<0.0001, one-way ANOVA.

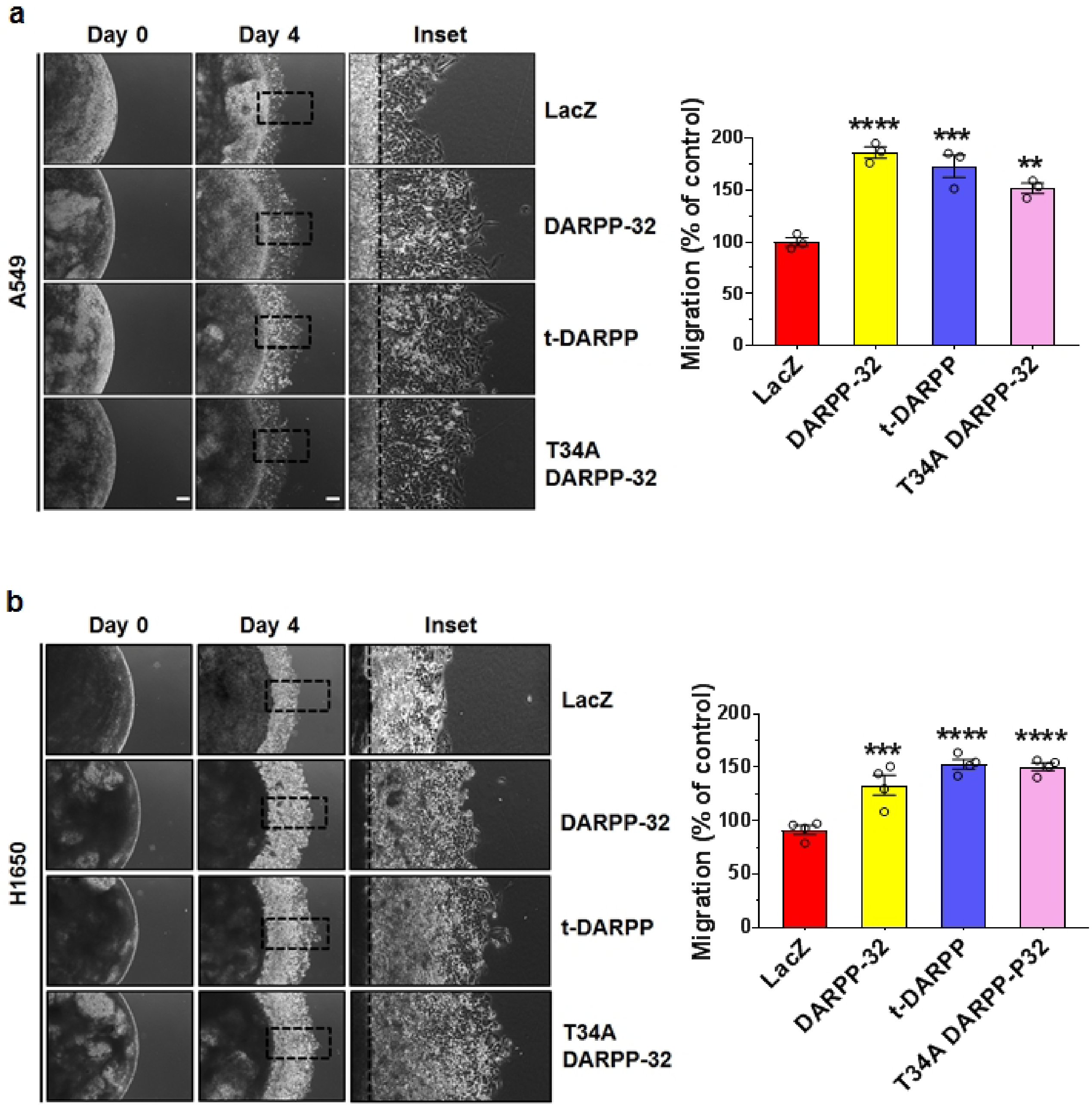

Supplementary Figure 4: DARPP-32 increases cell migration by spot assay. **a** A549 and **b** H1650 cells transduced with retrovirus encoding control, DARPP-32, t-DARPP or T34A DARPP-32 clones were mixed with Matrigel and spotted. Representative images depict one half of the spot at 0 and 4 days post-plating. Enlarged insets of day 4 images are depicted. The dashed line indicates the edge of the spot at day 0. Experiments were repeated at least three times. Scale bar, 200 µm. Distance travelled by the migratory cells were calculated using ImageJ software. Results represent mean ± SE. ***P*<0.01, ****P*<0.001 and *****P*<0.0001, one-way ANOVA.

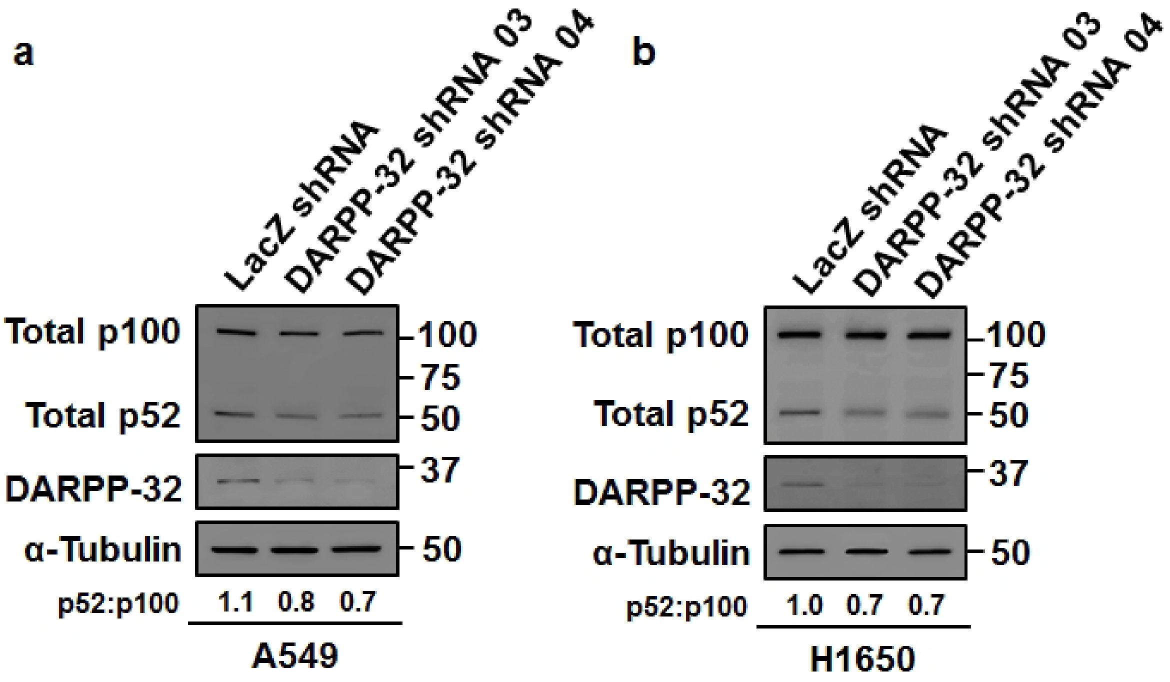

Supplementary Figure 5: Knockdown of DARPP-32 reduces p52 to p100 ratio. **a** DARPP-32-depleted unfractionated whole cell lysate of A549 and **b** H1650 cells were subjected to immunoblot analysis using antibodies against total p100, total p52, DARPP-32 and α-tubulin (loading control). Densitometry of the depicted relevant bands was performed using ImageJ software. The ratio of p52 to p100 is shown in the bottom panel. All immunoblots are representative of three independent experiments. Uncropped images of depicted immunoblots are shown in Supplementary Fig. 20c and 20d.

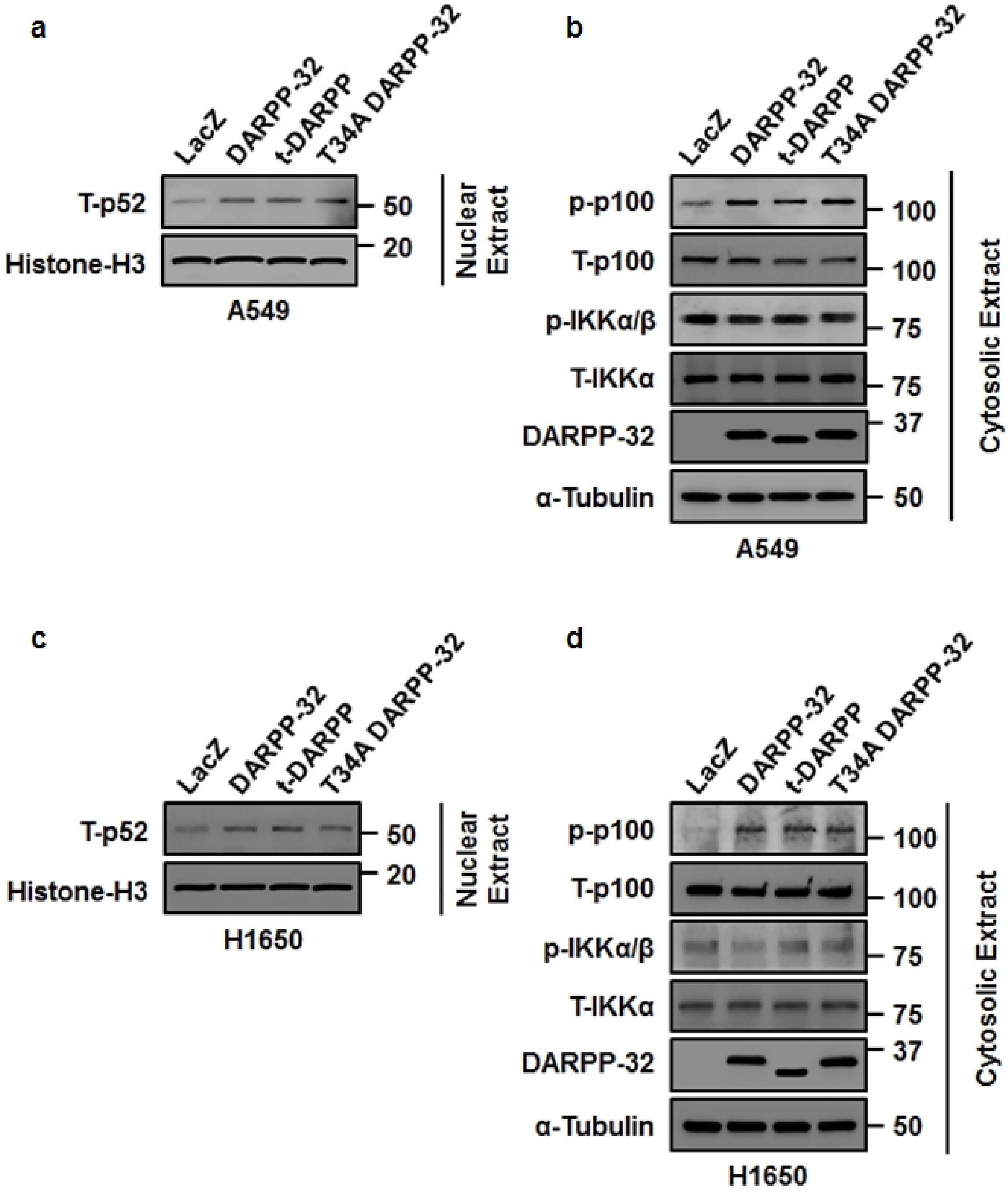

Supplementary Figure 6: DARPP-32 positively regulates non-canonical NF-κB2 signaling. **a** Nuclear and **b** cytosolic fractions of A549 cells overexpressing LacZ control, DARPP-32, t-DARPP or T34A DARPP-32 clones were immunoblotted with antibodies against total p52 (T-p52), Histone H3 (loading control), phosphorylated p100 (p-p100), total p100 (T-p100), phosphorylated IKKα/β (p-IKKα/β), total IKKα (T-IKKα), DARPP-32 and α-tubulin (loading control). **c** Nuclear and **d** cytosolic fractions of H1650 cells overexpressing LacZ control, DARPP-32, t-DARPP or T34A DARPP-32 clones were subjected to western blotting using antibodies against total p52 (T-p52), Histone H3 (loading control), phosphorylated p100 (p-p100), total p100 (T-p100), phosphorylated IKKα/β (p-IKKα/β), total IKKα (T-IKKα), DARPP-32 and α-tubulin (loading control). All immunoblots are representative of three independent experiments. Uncropped images of depicted immunoblots are shown in Supplementary Fig. 21.

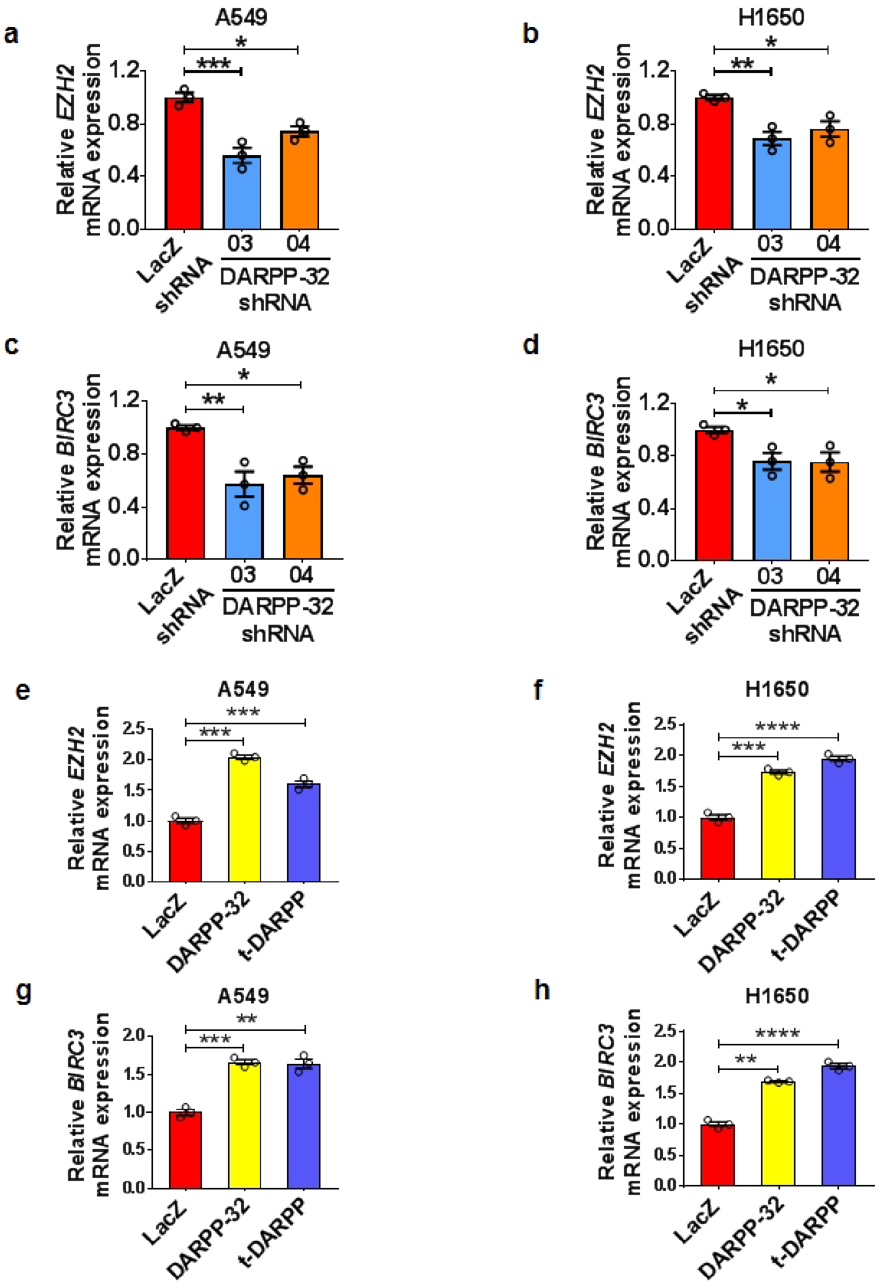

Supplementary Figure 7: DARPP-32 positively regulates the expression of NF-κB2 target genes. **a** Quantification of *EZH2* mRNA expression using qRT-PCR in DARPP-32-depleted A549 and **b** H1650 cell lines. **c** *BIRC3* mRNA expression was determined using qRT-PCR in DARPP-32 knockdown A549 and **d** H1650 cell lines. **e** The expression of *EZH2* mRNA was quantified using qRT-PCR in A549 and **f** H1650 cell lines overexpressing exogenous DARPP-32 isoforms. **g** Quantification of *BIRC3* mRNA expression using qRT-PCR in A549 and **h** H1650 cell lines overexpressing exogenous DARPP-32 isoforms.

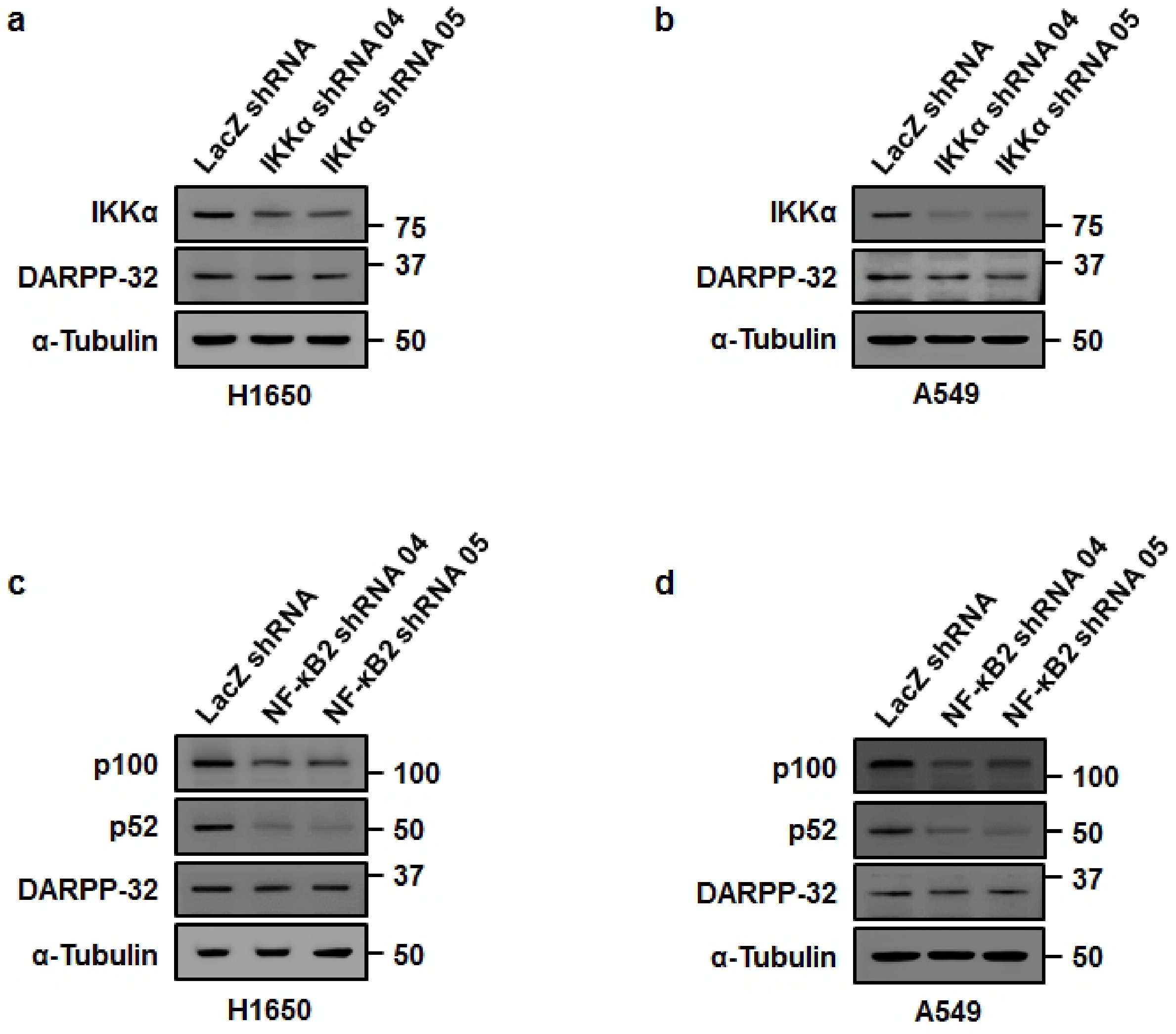

Supplementary Figure 8: Abrogation of IKKα and NF-κB2 expression in NSCLC cells. **a** IKKα-depleted H1650 and **b** A549 cells were immunoblotted using antibodies against total IKKα, DARPP-32 and α-tubulin (loading control). **c** H1650 and **d** A549 cells were transduced with lentivirus encoding NF-κB2 shRNAs. Cell lysates were immunoblotted using antibodies against total NF-κB2 (detects both p100 and p52 subunits), DARPP-32 and α-tubulin (loading control). All immunoblots are representative of three independent experiments. Uncropped images of depicted immunoblots are shown in Supplementary Fig. 22.

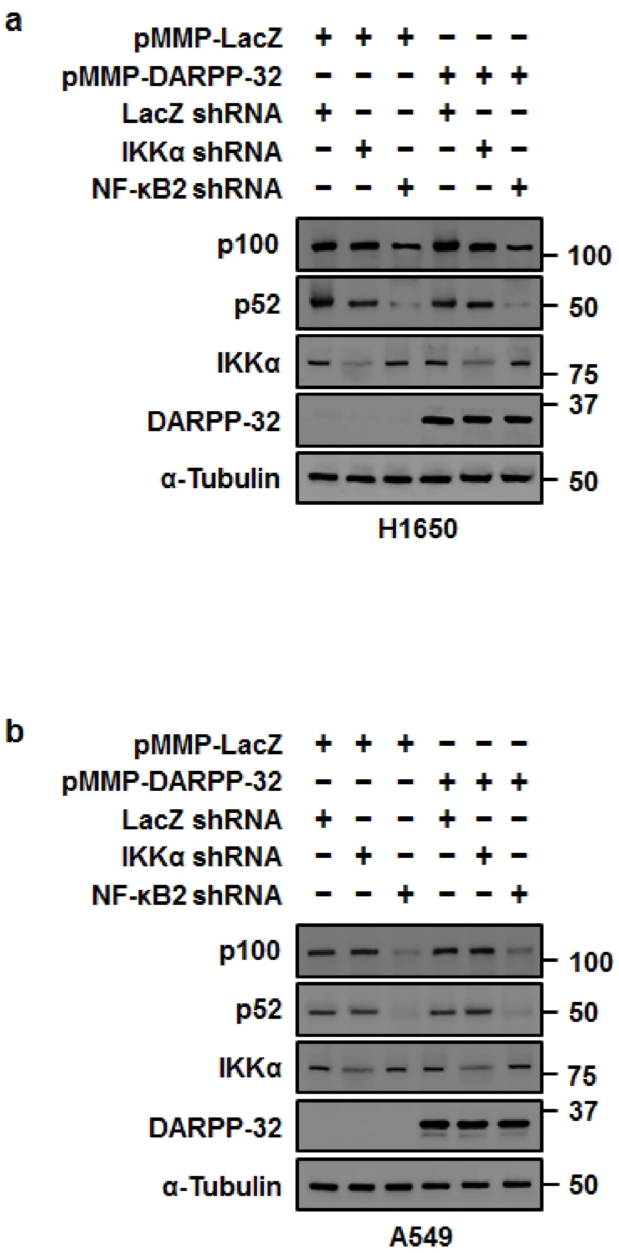

Supplementary Figure 9: Overexpression of DARPP-32 in IKKα- and NF-κB2-depleted NSCLC cells. **a** H1650 and **b** A549 cells were transduced with lentivirus encoding IKKα or NF-κB2 shRNAs and then transfected with control (pMMP-LacZ) or DARPP-32 overexpressing plasmids (pMMP-DARPP-32) for 48 h. Cell lysates were harvested and immunoblotted using antibodies against total NF-κB2 (detects both p100 and p52 subunits), total IKKα, DARPP-32 and α-tubulin (loading control). All immunoblots are representative of three independent experiments. Uncropped images of depicted immunoblots are shown in Supplementary Fig. 23.

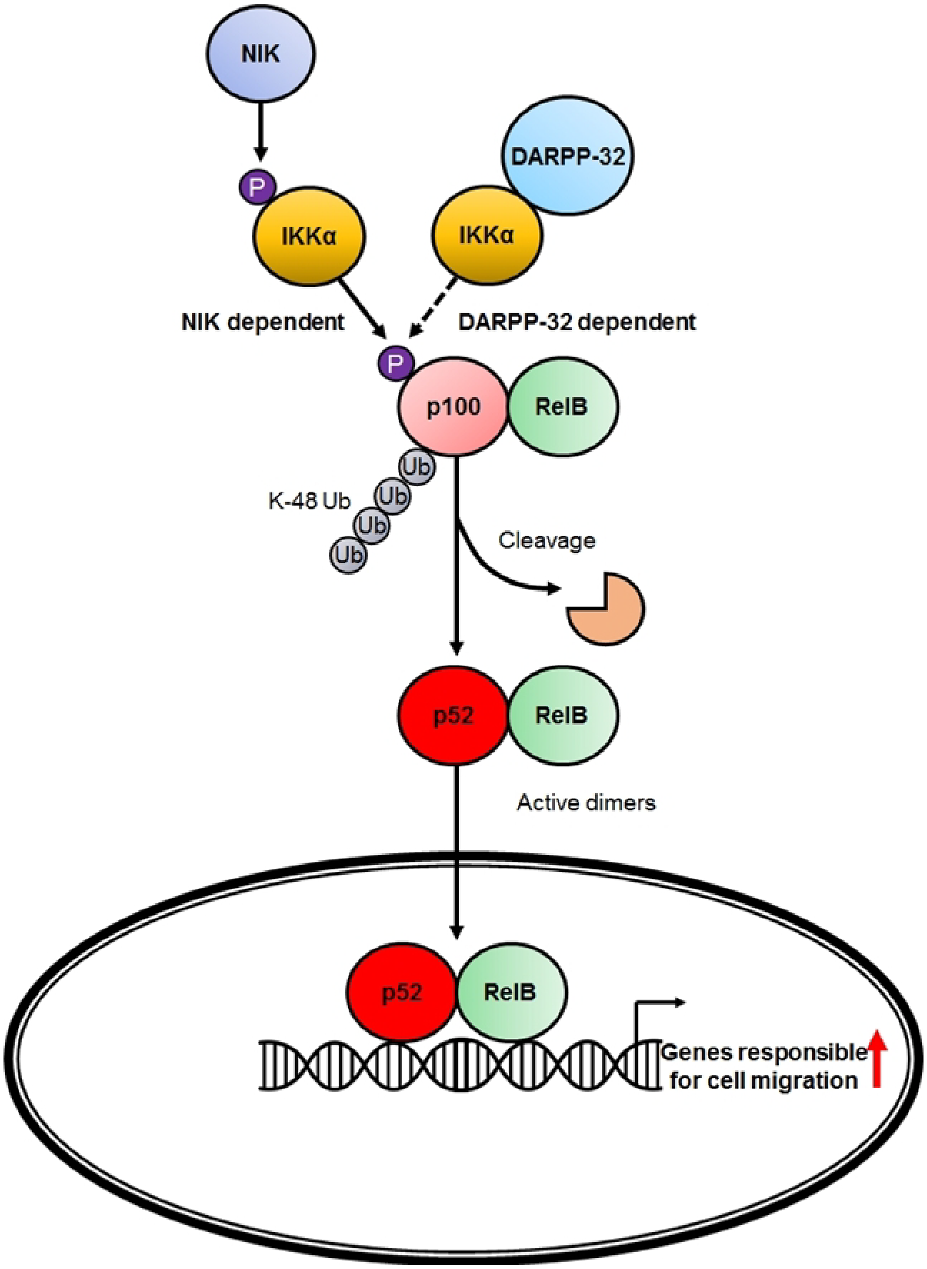

Supplementary Figure 10: DARPP-32 promotes lung cancer cell migration through regulation of non-canonical NF-κB2 signaling. Upon stimulation, NF-κB inducing kinase (NIK) phosphorylates IKKα, which facilitates IKKα-mediated phosphorylation of p100 at S866/870. This phosphorylation event initiates subsequent ubiquitination at K48 and partial degradation of p100 by the proteasome to form p52. RelB-p52 heterodimers then translocate to the nucleus to regulate gene expression, including genes involved in cellular migration. Our data demonstrating a physical interaction between IKKα and DARPP-32 suggests IKKα-dependent phosphorylation of p100 may be directly or indirectly mediated by DARPP-32 through an NIK-independent mechanism (represented in dotted line) in human NSCLC cells.

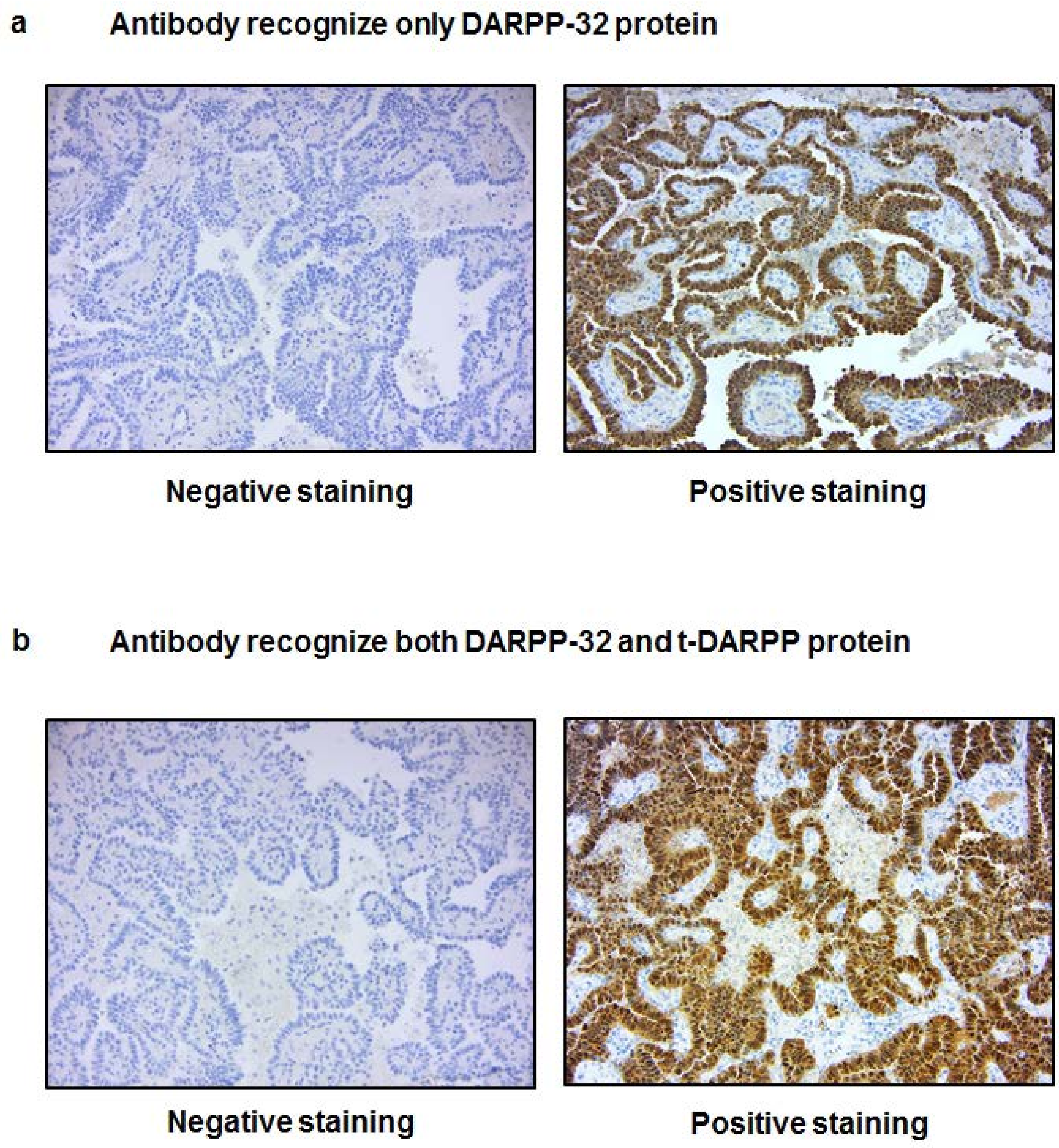

Supplementary Figure 11: Antibody specificity controls for each of the DARPP-32 antibodies used in the IHC studies. **a** IHC was performed using no primary antibody (i.e. negative straining) and using an N-terminal DARPP-32 antibody that exclusively detects DARPP-32 (i.e. positive staining). **b** IHC was performed using no primary antibody (i.e. negative straining) and using a C-terminal DARPP-32 antibody that detects both DARPP-32 and t-DARPP (i.e. positive staining). Serial sections of the same reference human lung cancer tissue were used for all the antibody specificity controls shown in this figure.

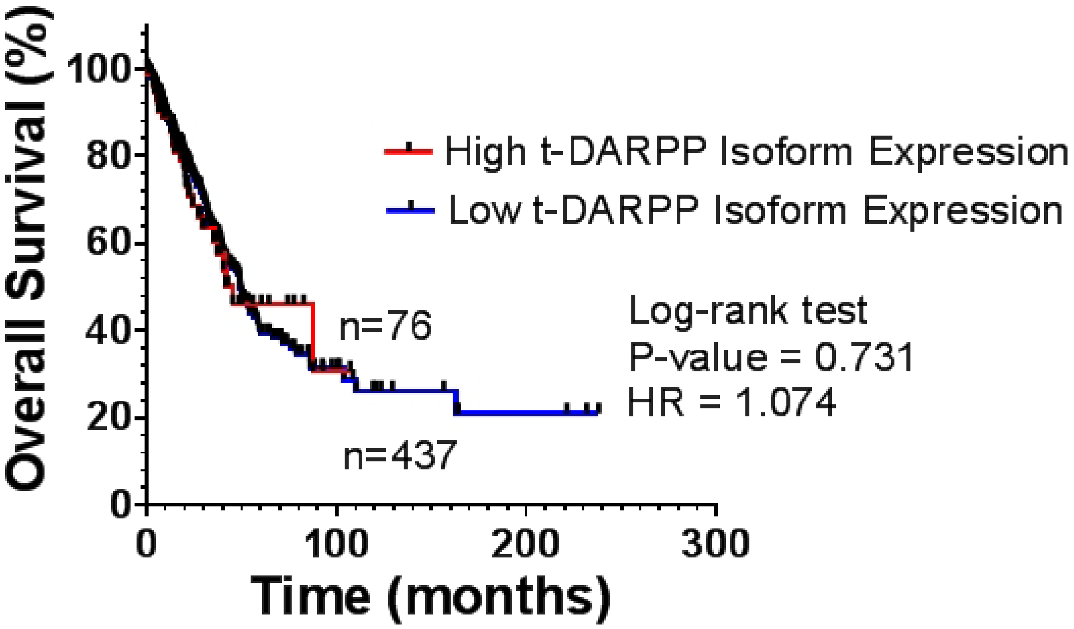

Supplementary Figure 12: Expression of total t-DARPP isoform does not correlate with lung adenocarcinoma patient survival. Kaplan Meier plot showing overall survival within the total cohort of 513 NSCLC patients based on the expression of t-DARPP isoform. The normalized read count for t-DARPP isoform was obtained from The Cancer Genome Atlas dataset (TCGA). The difference between the two groups was calculated using Log-rank (Mantel-Cox) test. HR: hazard ratio.

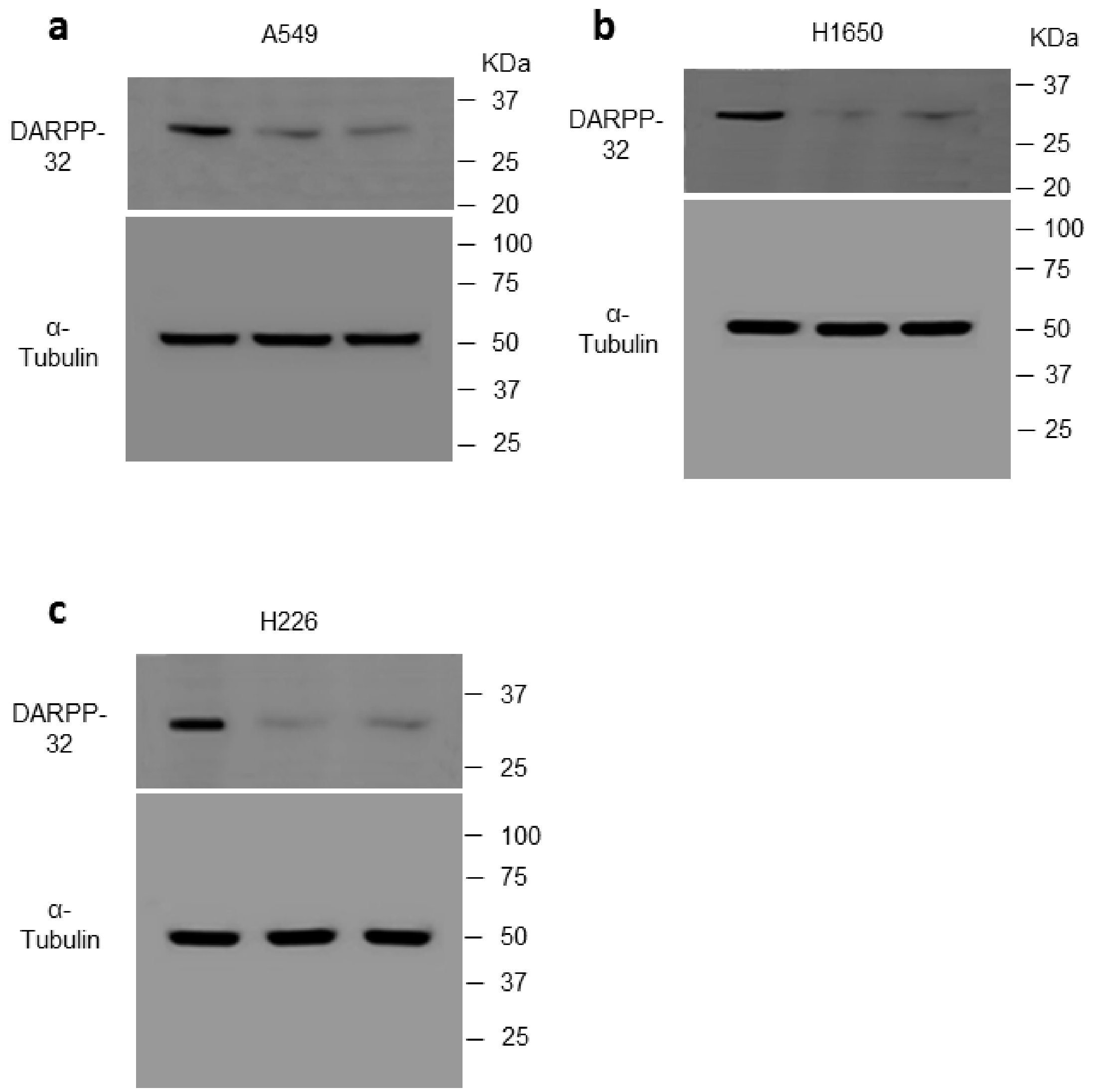

Supplementary Figure 13: Full-sized scans of immunoblots in Figure 1a, Figure 1b, and Figure 1c.

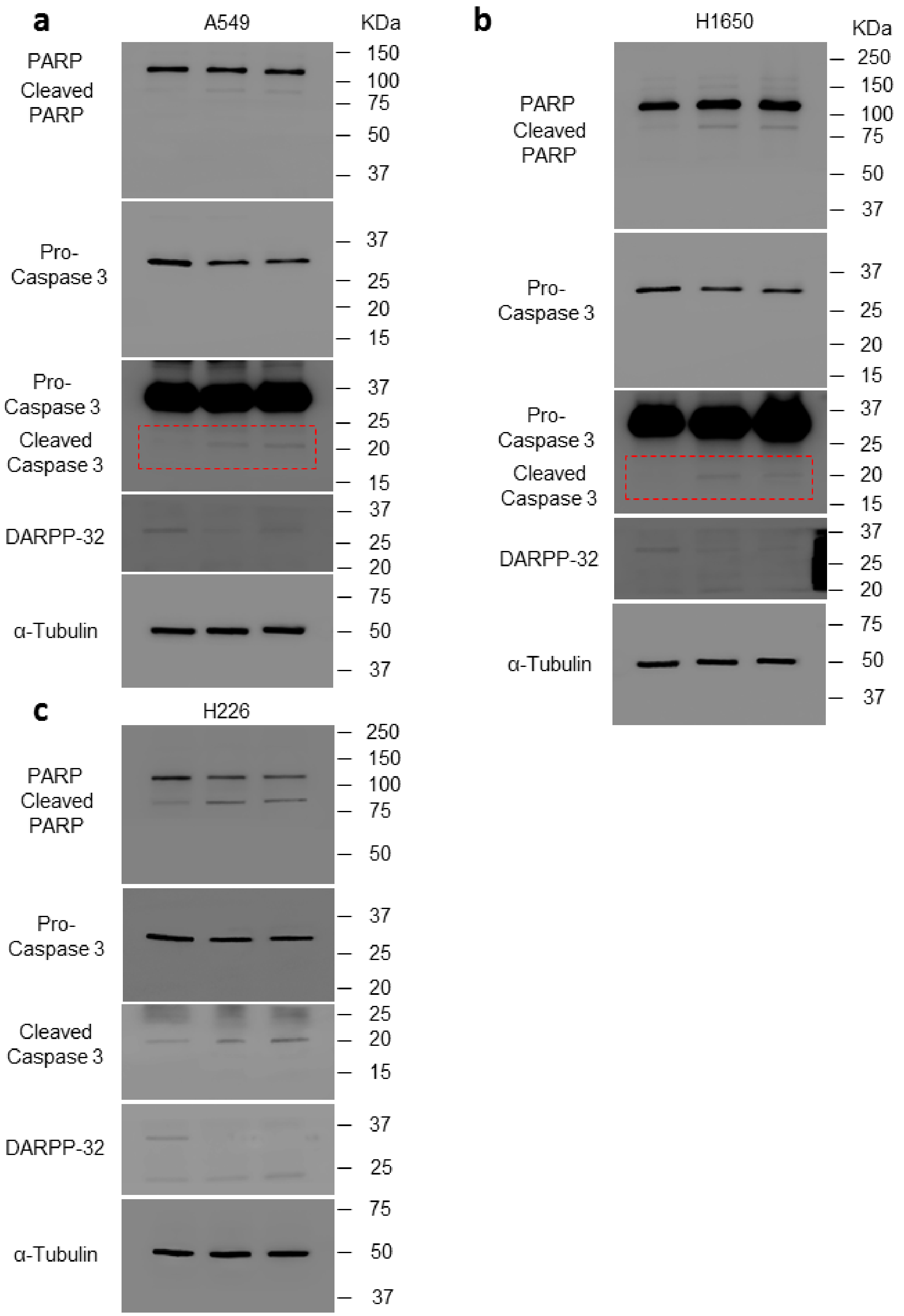

Supplementary Figure 14: Full-sized scans of immunoblots in Figure 1g, Figure 1h, and Figure 1i.

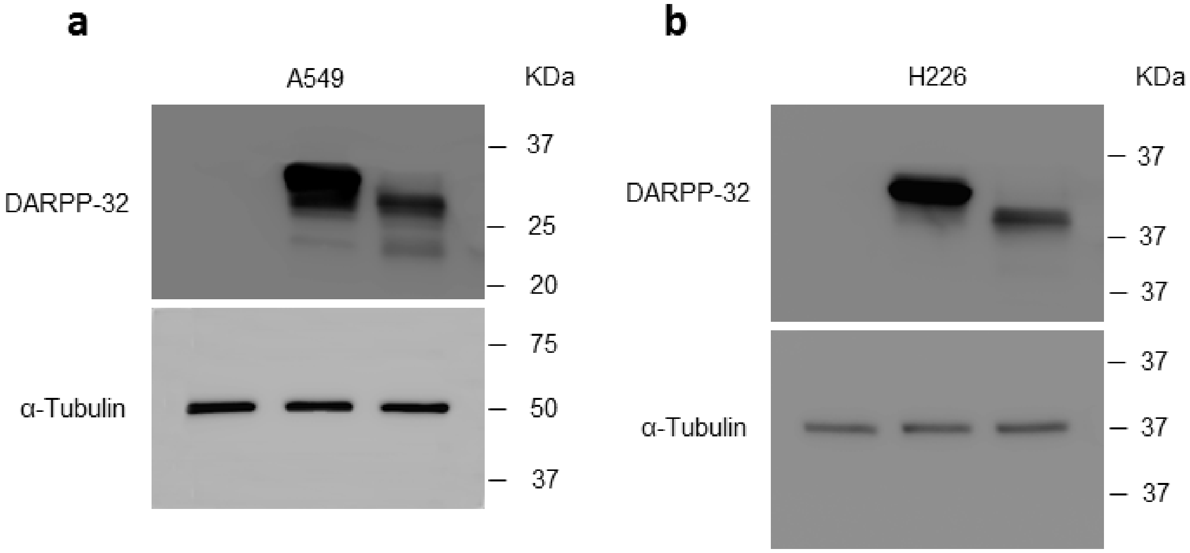

Supplementary Figure 15: Full-sized scans of immunoblots in Figure 1j and Figure 1k.

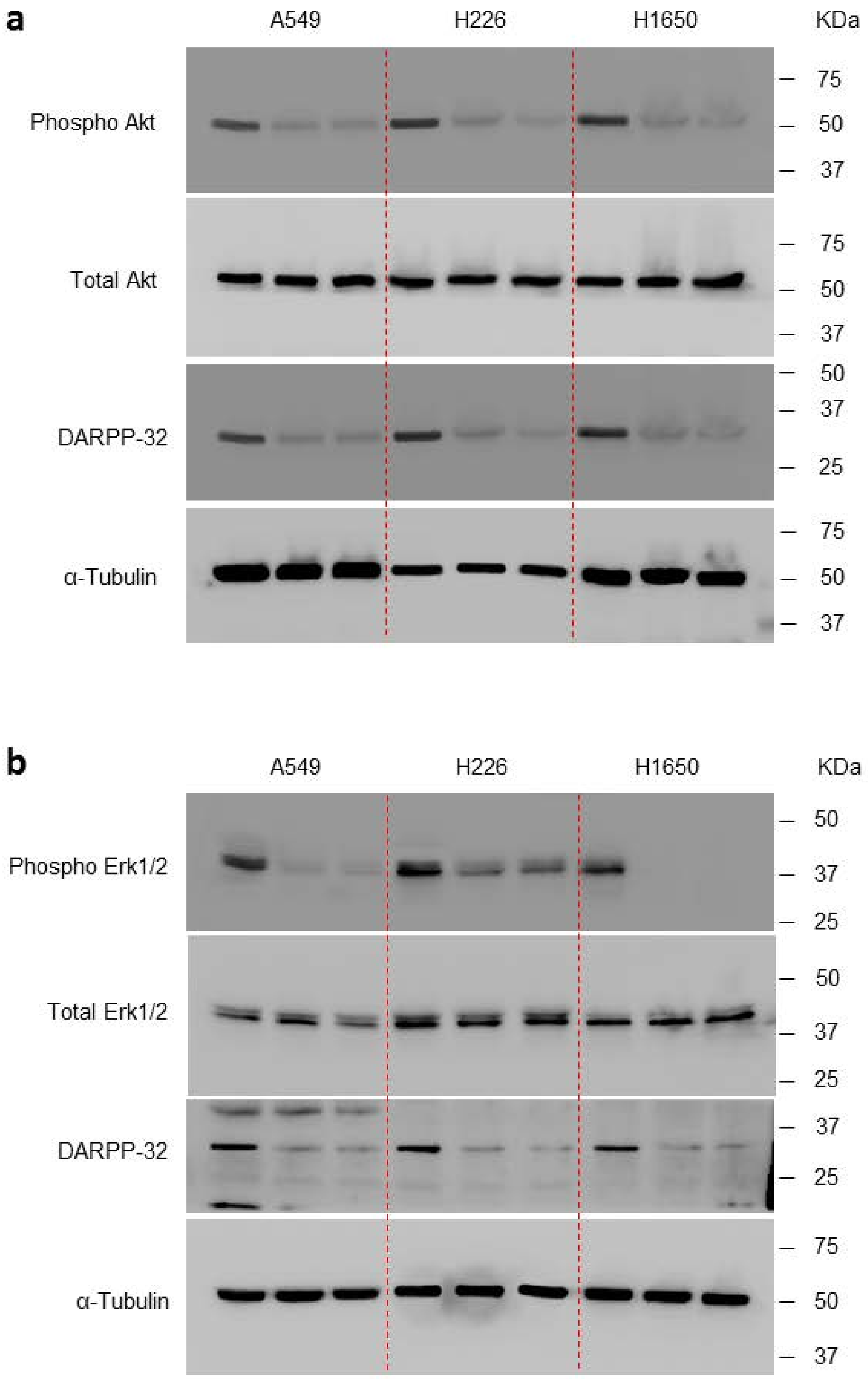

Supplementary Figure 16: Full-sized scans of immunoblots in Figure 2a and Figure 2b.

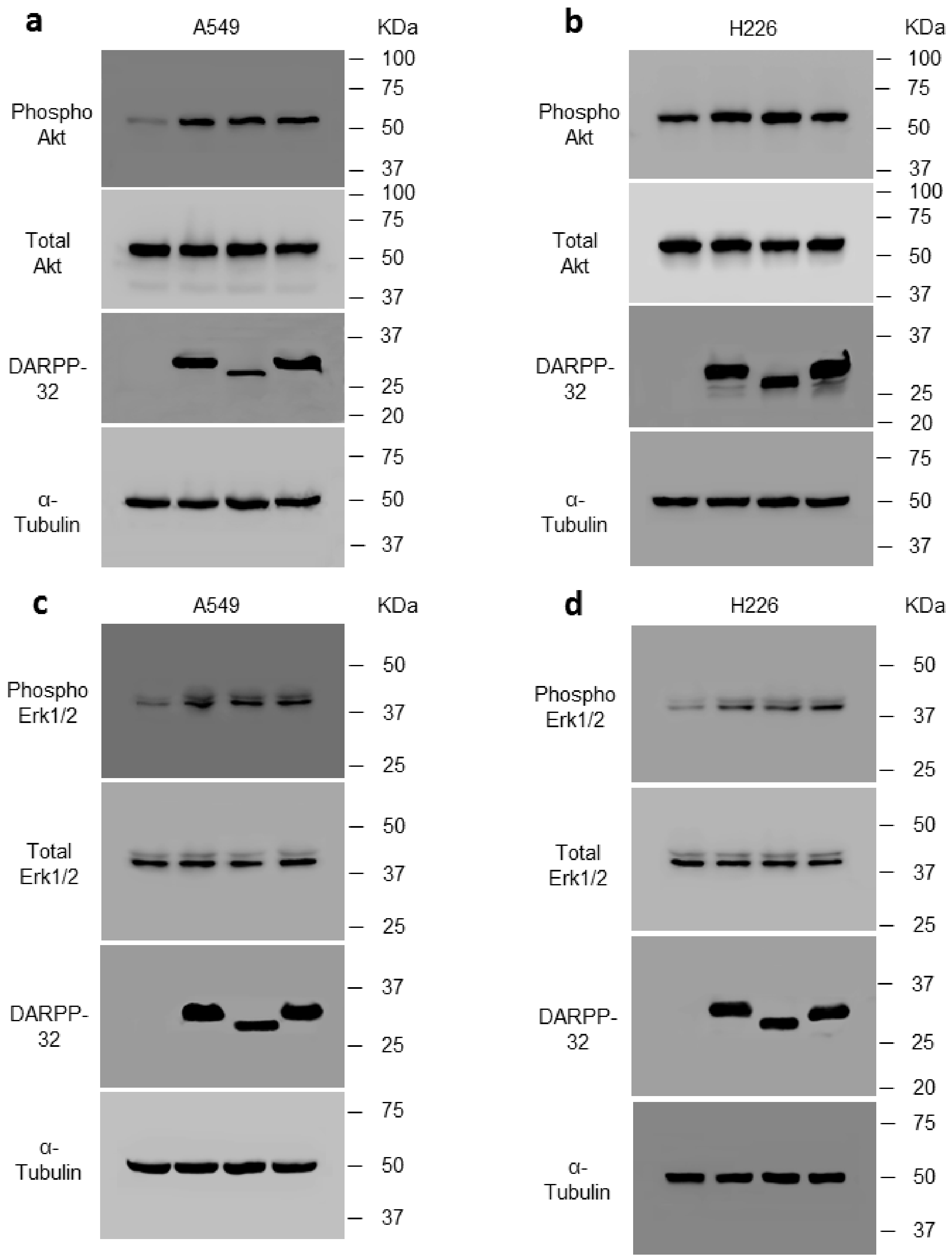

Supplementary Figure 17: Full-sized scans of immunoblots in Figure 2c and Figure 2d.

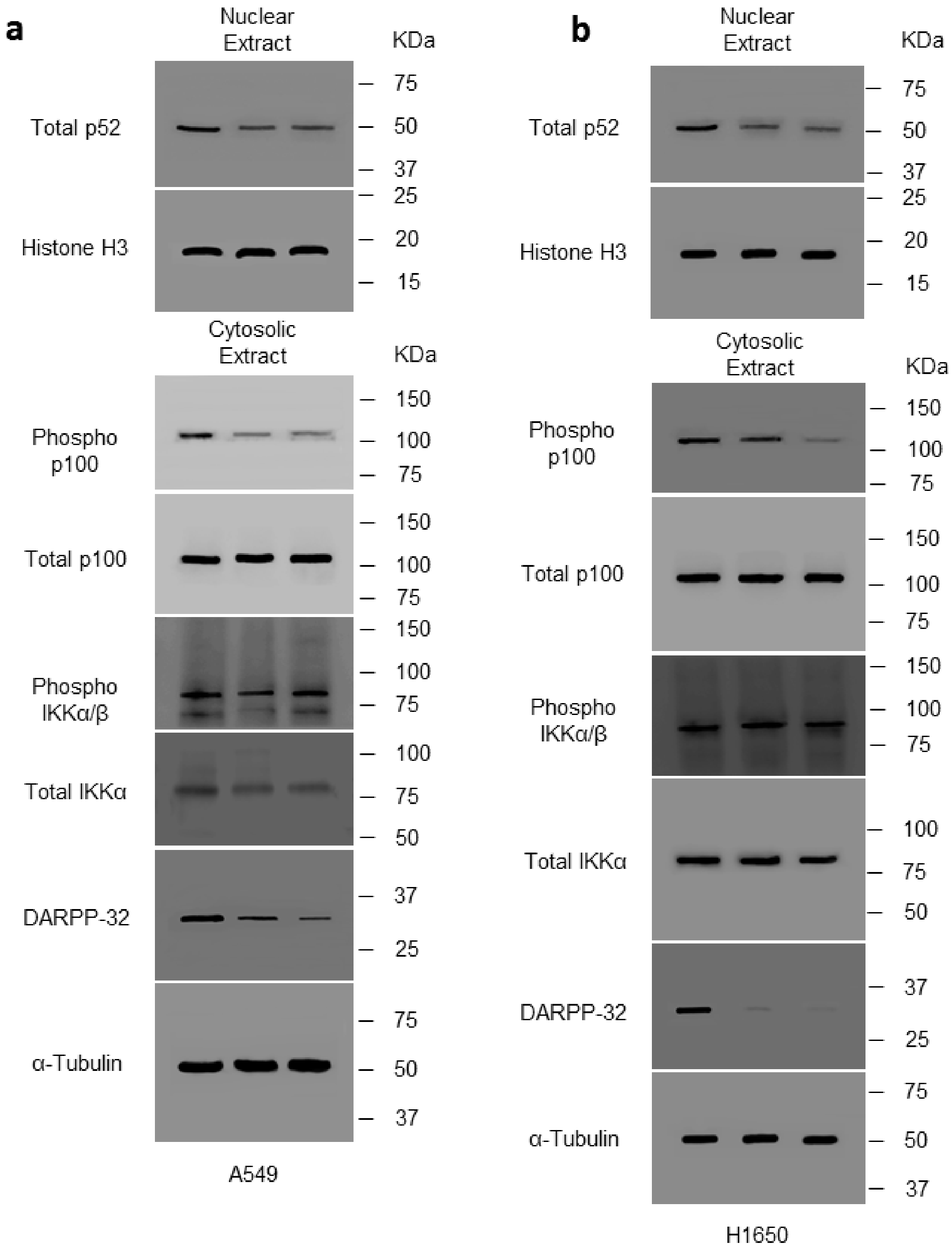

Supplementary Figure 18: Full-sized scans of immunoblots in Figure 4a and Figure 4b.

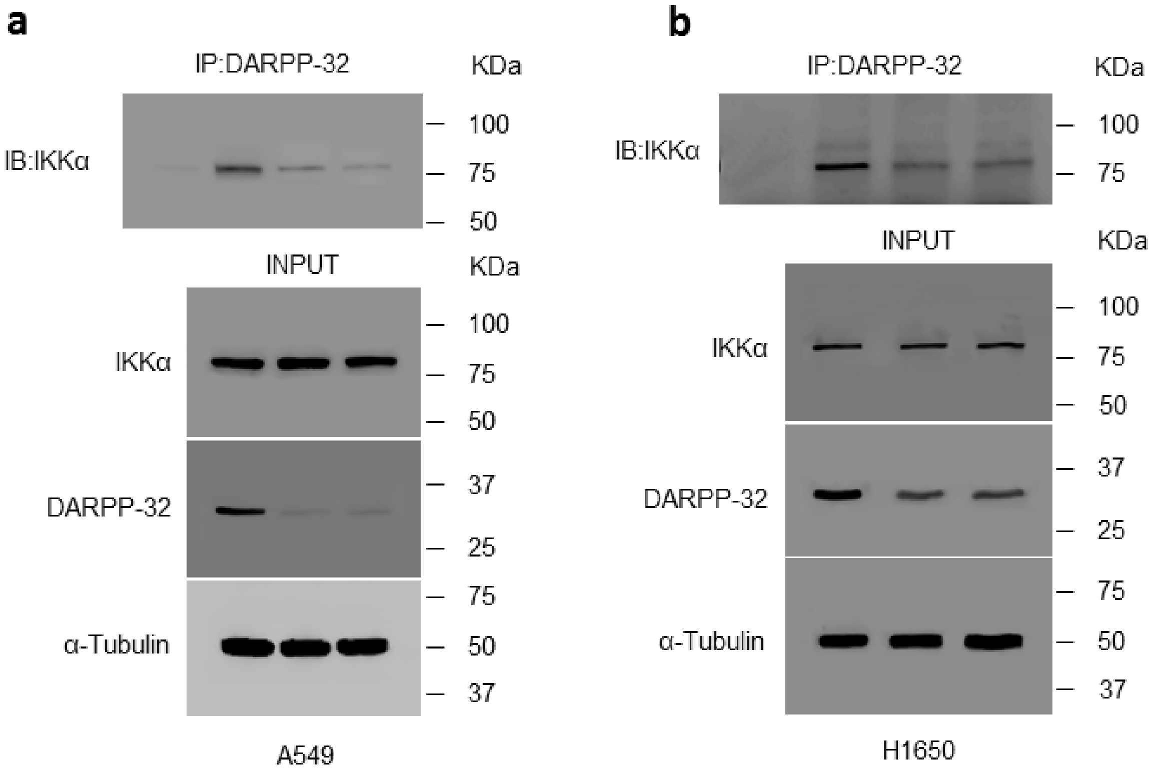

Supplementary Figure 19: Full-sized scans of immunoblots in Figure 4e and Figure 4f.

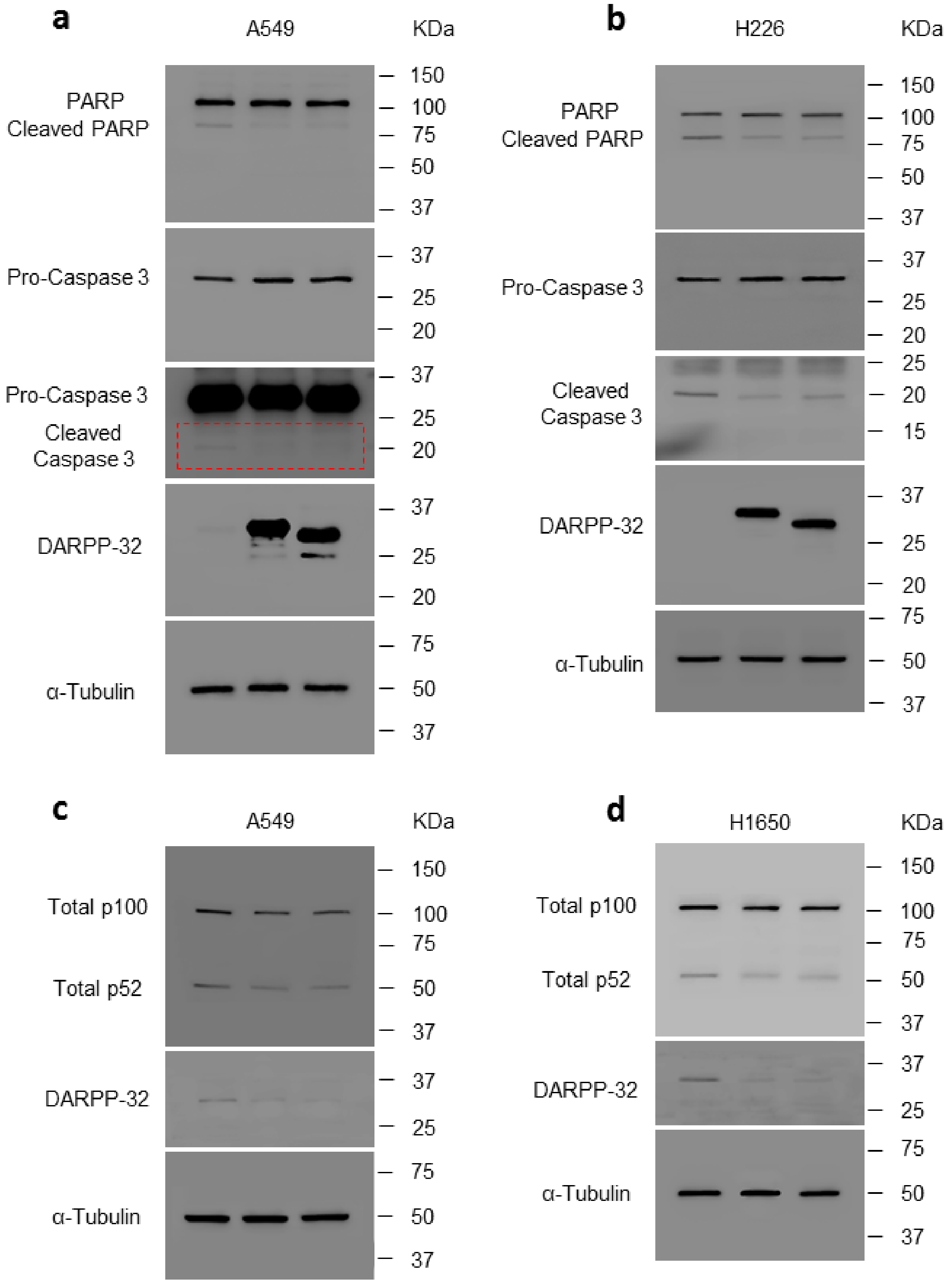

Supplementary Figure 20: Full-sized scans of immunoblots in Supplementary Figure 1c, 1d, 5a, and 5b.

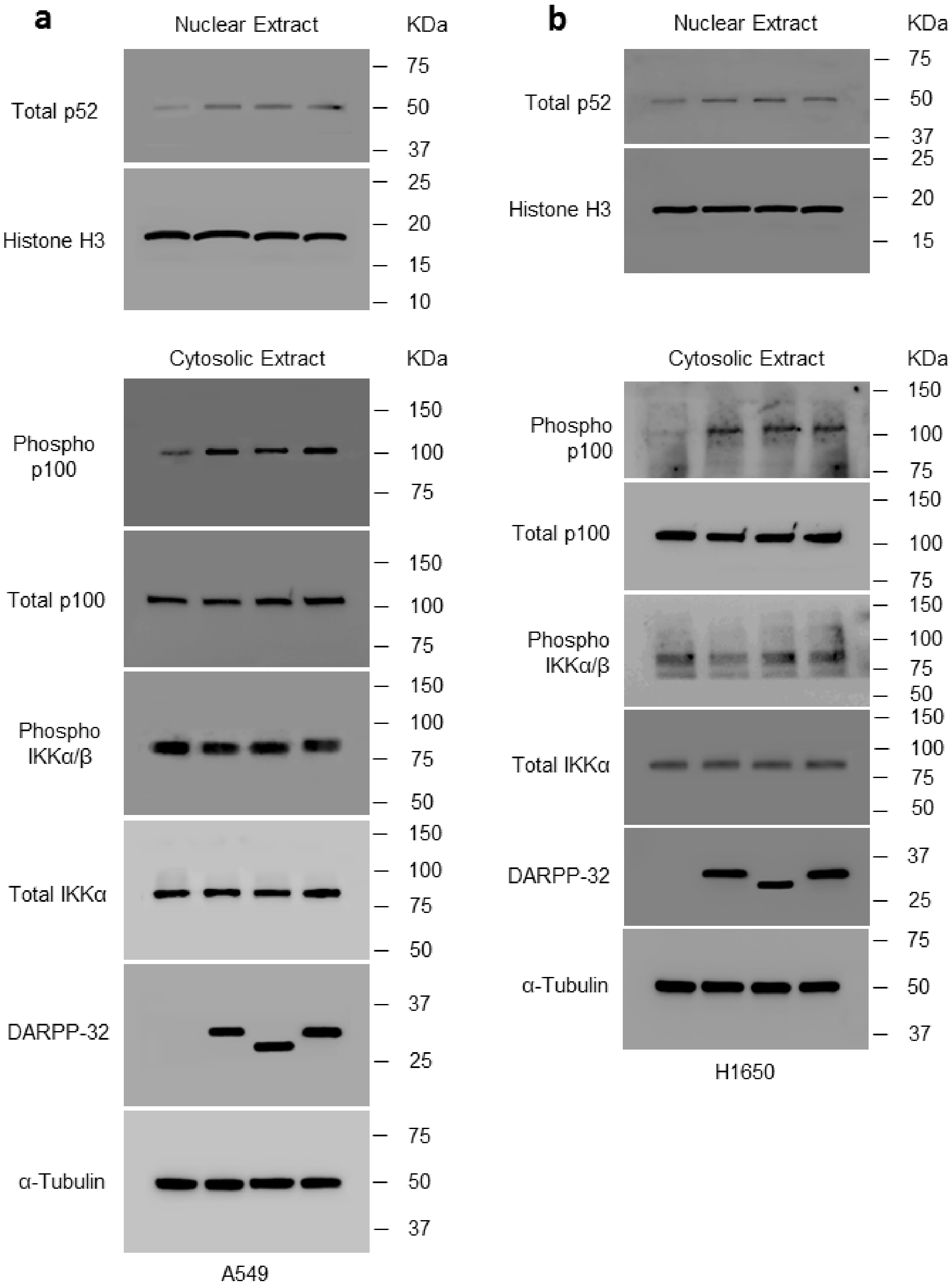

Supplementary Figure 21: Full-sized scans of immunoblots in Supplementary Figure 6a, 6b, 6c, and 6d.

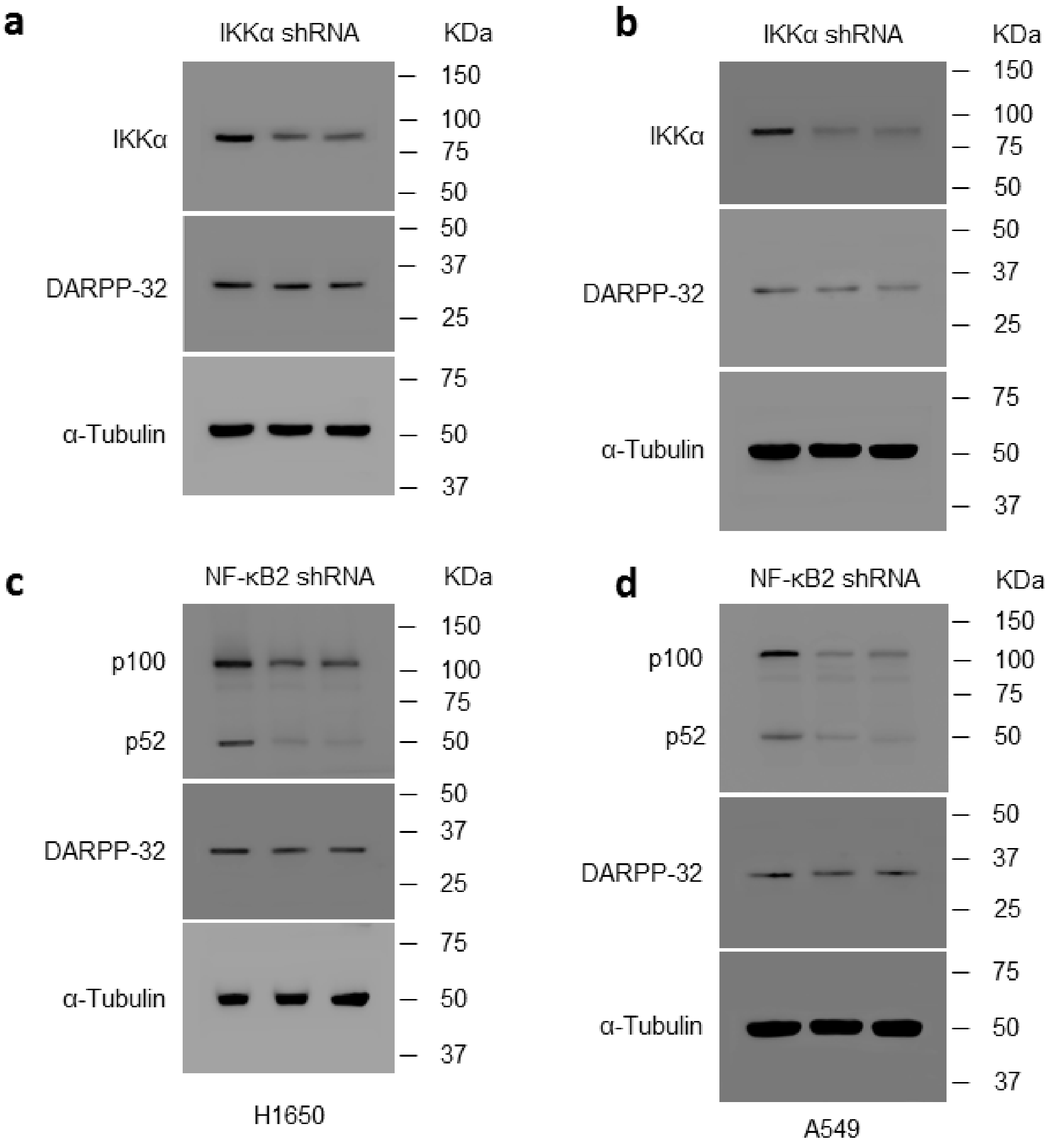

Supplementary Figure 22: Full-sized scans of immunoblots in Supplementary Figure 8a, 8b, 8c, and 8d.

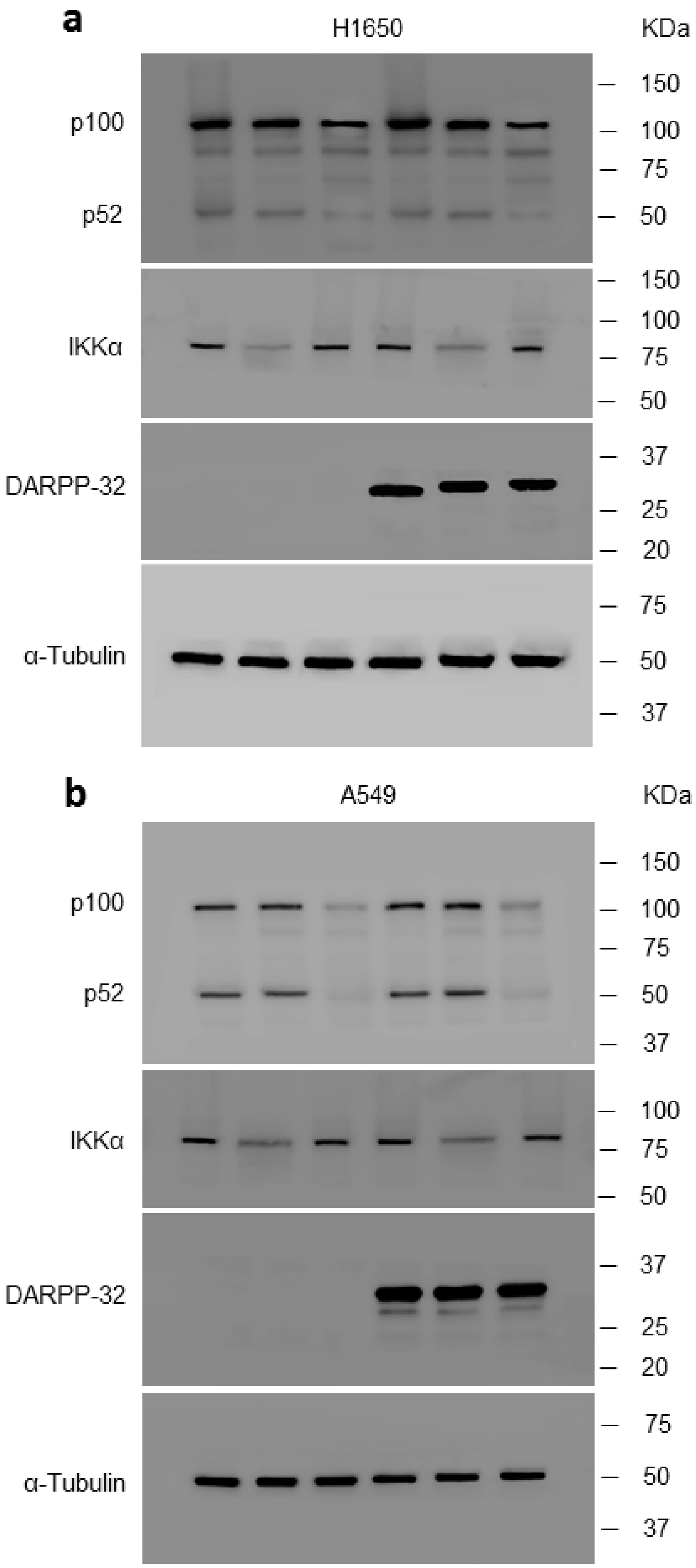

Supplementary Figure 23: Full-sized scans of immunoblots in Supplementary Figure 9a and 9b.

Supplementary Table 1: Clinical characteristics of 62 lung adenocarcinoma patients corresponding to tissue specimens used to evaluate t-DARPP expression by immunohistochemistry

CASE	GENDER	AGE	TUMOR	STAGE	DARPP-32 STAINING			DARPP-32 & t-DARPP STAINING		
					Tumor Cell (%)	Intensity	IR Score	Tumor Cell (%)	Intensity	IR Score
1	MALE	61	T3	IIIA	90	3	270	100	3	300
2	MALE	64	T3	IIIA	50	2	100	75	3	225
3	MALE	65	T2	IIIA	0	0	0	0	0	0
4	FEMALE	66	T1	IIB	0	0	0	1	2	2
5	MALE	72	T1	IIIA	60	3	180	70	3	210
6	FEMALE	82	T4	IV	90	3	270	95	3	285
7	FEMALE	80	T3	IIIB	95	3	285	95	3	285
8	FEMALE	60	T1	IIIA	100	3	300	100	3	300
9	FEMALE	72	T1	IIIA	5	3	15	5	3	15
10	MALE	66	T1	IIIA	0	0	0	2	3	6
11	MALE	71	T2	IIIA	1	1	1	5	2	10
12	FEMALE	71	T2	IIIA	100	3	300	100	3	300
13	FEMALE	72	T3	IIIA	5	3	15	7	3	21
14	MALE	72	T2	IIIA	95	3	285	95	3	285
15	MALE	64	T1	IIIA	1	3	3	2	3	6
16	MALE	76	T3	IIIB	100	3	300	100	3	300
17	FEMALE	51	T3	IIIA	60	3	180	80	3	240
18	MALE	50	T2	IIIA	100	3	300	100	3	300
19	FEMALE	52	T1	IIIA	70	3	210	80	3	240
20	FEMALE	53	T3	IIIA	5	3	15	10	3	30
21	MALE	76	T3	IIIB	80	3	240	80	3	240
22	FEMALE	68	T4	IV	80	3	240	90	3	270
23	FEMALE	47	T3	IIIA	20	3	60	30	3	90
24	FEMALE	59	T4	IIIB	0	0	0	0	0	0
25	FEMALE	36	T2	IIIA	60	3	180	60	3	180
26	FEMALE	53	T1	IIIA	30	3	90	60	3	180
27	MALE	66	T3	IIIA	70	3	210	80	3	240
28	MALE	73	T4	IV	70	3	210	85	3	255

CASE	GENDER	AGE	TUMOR	STAGE	DARPP-32 STAINING			DARPP-32 & t-DARPP STAINING		
					Tumor Cell (%)	Intensity	IR Score	Tumor Cell (%)	Intensity	IR Score
29	FEMALE	56	T1	IIIA	95	3	285	95	3	285
30	FEMALE	42	T3	IV	0	0	0	0	0	0
31	MALE	71	T2	IIIA	70	3	210	90	3	270
32	MALE	37	T4	IV	100	3	300	100	3	300
33	MALE	57	T1	IIIA	90	3	270	100	3	300
34	MALE	66	T3	IIIA	20	3	60	40	3	120
35	MALE	54	T4	IIIB	30	3	90	30	3	90
36	MALE	65	T3	IIIA	60	3	180	100	3	300
37	MALE	55	T2	IIIA	0	0	0	1	3	3
38	MALE	62	T1	IIIA	70	3	210	85	3	255
39	MALE	49	T2	IIIA	80	3	240	90	3	270
40	FEMALE	72	T2	IIIA	80	3	240	100	3	300
41	MALE	65	T3	IIIA	90	3	270	100	3	300
42	MALE	67	T2	IIIA	0	0	0	1	3	3
43	MALE	69	T1	IIIA	0	0	0	0	0	0
44	MALE	56	T4	IIIB	0	0	0	1	3	3
45	MALE	67	T1	IIIA	10	3	30	10	3	30
46	MALE	69	T2	IIIA	1	3	3	1	3	3
47	MALE	76	T4	IV	1	2	2	3	3	9
48	MALE	72	T4	IV	100	3	300	100	3	300
49	FEMALE	60	T1	IIIA	90	3	270	90	3	270
50	FEMALE	70	T1	IIIA	10	3	30	60	3	180
51	MALE	60	T3	IIIA	60	3	180	60	3	180
52	FEMALE	60	T2	IIIA	10	3	30	10	3	30
53	FEMALE	73	T2	IIIA	60	3	180	70	3	210
54	MALE	59	T3	IIIA	0	0	0	0	0	0
55	FEMALE	63	T3	IIIA	95	3	285	95	3	285
56	MALE	63	T3	IIIB	<1	3	3	1	3	3
57	MALE	36	T3	IIIA	0	0	0	0	0	0
58	FEMALE	65	T2	IIIA	0	0	0	1	3	3
59	MALE	55	T2	IIIA	10	3	30	30	3	90

CASE	GENDER	AGE	TUMOR	STAGE	DARPP-32 STAINING			DARPP-32 & t-DARPP STAINING		
					Tumor Cell (%)	Intensity	IR Score	Tumor Cell (%)	Intensity	IR Score
60	MALE	53	T3	IIIA	<1	3	3	1	3	3
61	MALE	65	T1	IIIA	0	0	0	1	3	3
62	MALE	50	T4	IIIA	30	3	90	60	3	180
